# Morphological studies on the prehatching development of the glandular stomach of Japanese quails using light, electron, and fluorescent microscopy

**DOI:** 10.1038/s41598-023-45355-1

**Published:** 2023-10-23

**Authors:** Wafaa Gaber, Heba Mostafa, Yousria A. Abdel-Rahman, Hanan H. Abd El-Hafeez

**Affiliations:** 1 Department of Anatomy and Embryology, Faculty of Veterinary Medicine, Assiut University, Assiut, Egypt; 2 Department of Cell and Tissues, Faculty of Veterinary Medicine, Assiut University, Assiut, Egypt

**Keywords:** Developmental biology, Structural biology

## Abstract

The development of the glandular stomach was studied using light, electron, and fluorescent microscopy. The research used 130 Japanese quail eggs from the second to the seventeenth days of incubation.The proventriculus could be distinguished on the3rd day. Its wall consisted of four tunics: tunica mucosa, very thin tunica submucosa, tunica muscularis, and outermost tunica serosa. Mucosal folds appeared on the 8th day. The luminal epithelium was pseudostratified columnar in type and transformed into simple columnar by the 10th day. The mucosal papillae emerged on the 11th day, spiraled on the 15th day, and had a distinct whorled look by the 17th day. Two types of proventricular glands were recognized: compound tubuloalveolar and simple tubular glands. Both types were situated within the tunica mucosa. On the 4th day, the compound glands emerged as evaginations of the lining epithelium. It began to branch on the 8th day and became well established by the 11th day. The simple glands appeared on the 11th day as localized down-growths of the luminal epithelium forming solid cords. On the 15th day, many of them showed complete canalization. On the 8th day, the muscular coat was differentiated into the lamina muscularis mucosae and tunica muscularis.

## Introduction

In developmental research, various animal models, including avian species, are currently employed. Among avian species, the chick embryo serves as a paradigm for developmental biologists. The Japanese quail (*Coturnix japonica*) was recently introduced as a popular model for embryological research^1^. Several aspects account for the utility of this bird. First of all, it has become important to the economy as a species that can be farmed and makes tasty eggs and meat^2^. Second, it is a great lab animal because it doesn’t need much care, is small (80–300 g), has a fast rate of reproduction (three to four babies a year), and is resistant to diseases^3–6^. Its short incubation period and easy access to its egg makes it a great model for developmental biology. Its early maturity (it can reproduce at 6 weeks old) make it useful for studying ageing and disease^7–12^.

The avian stomach is composed of two basic morphologically and physiologically distinct regions: a glandular portion, or proventriculus, and a muscular portion, or gizzard. Both parts are characterized by great morphological and functional variability, both between and within species. It is mostly influenced by accessibility, with the amount and kind of food changing over time^13,14^. The proventriculus is a fusiform structure found in most birds between the esophagus and the gizzard. Its size varies in different kinds of birds, where it appears small in graminivorous birds and huge and distensible in carnivorous birds^15^. The proventriculus' major purpose is to produce hydrochloric acid and pepsinogen into the digestive compartments, which will churn the ingested materials via muscular mechanisms^13,14,16^.

The quail stomach consists of two basic compartments having the same morphology and function as those of other birds^17^. Many text books^18–23^, as well as numerous publications^24–31^, have detailed the histomorphology and development of the glandular stomach of diverse avian species. The histomorphology and development of the avian digestive system has been described in a number of published works^32–42^.

Except for Attia’s^43^ and Soliman et al.^30^ studies on the histogenesis of the stomach of pre-hatching quails, there has been little developmental research on the glandular stomach of quails. However, these researchers noted differences in some ages. Therefore, the purpose of this study is to investigate and provide detailed information about the gross, histological, and histochemical changes of the proventriculus at different stages of development during the pre-hatching period of Japanese quails, as well as the proventriculus’ fine structure, using a, using a light, electron (scanning and transmission), and fluorescent microscope.

## Results

### Two-days embryos

**Figure 1:**
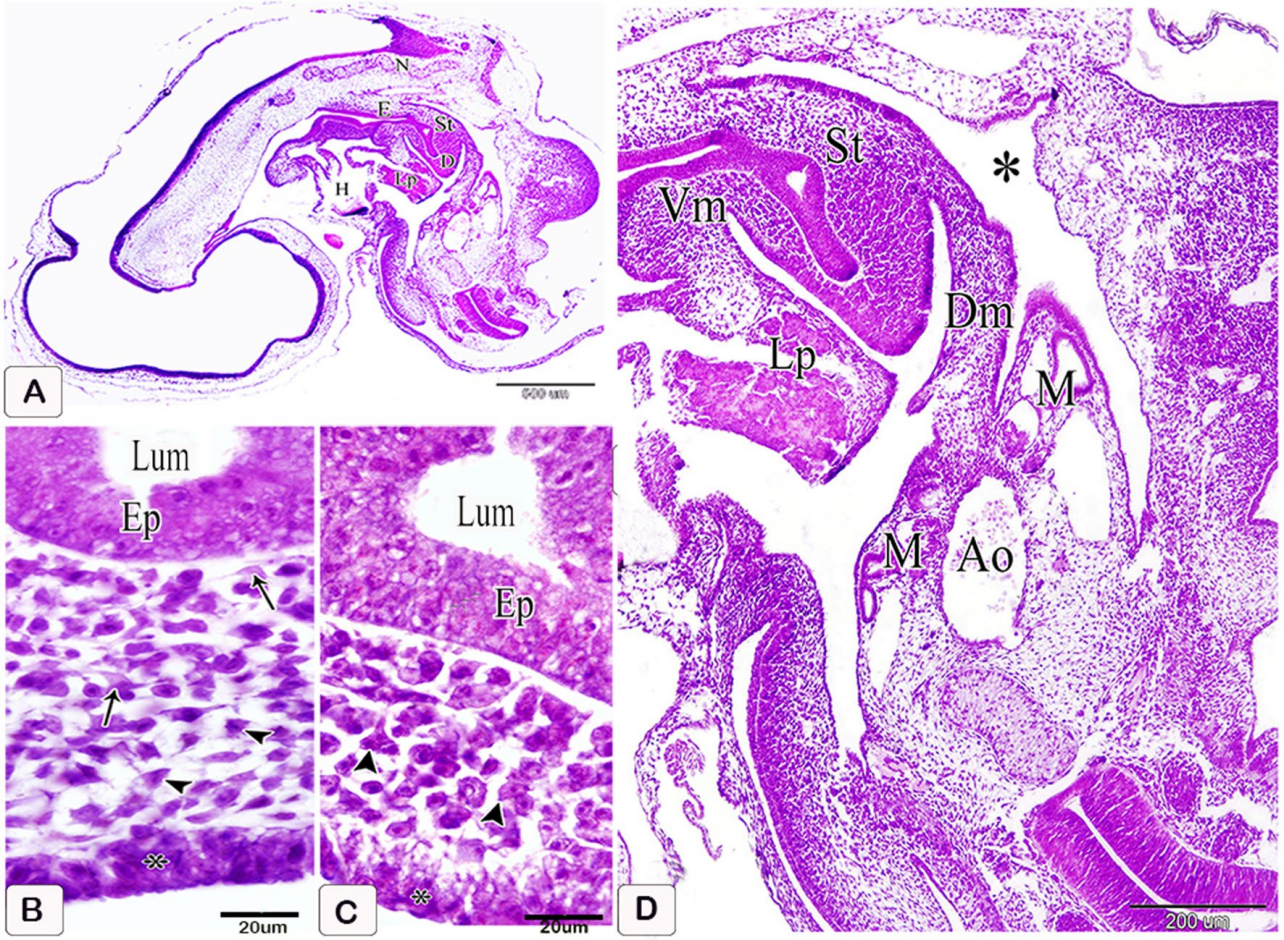
Photomicrographs of paraffin sections in the stomach primordium of a 2-day-old quail embryo stained with hematoxylin and eosin. (**A**) Photomicrograph of a sagittal section of a 2-day-old quail embryo showing the stomach primordium (St), esophgus (E), duodenum (D), notochord (N), liver primordium (Lp), and heart. (**B** and **C**) Photographs of sections in the stomach primordium of a 2-day-old quail embryo showing the structure of its wall dorsally (b) and ventrally (C). Epithelium (Ep), star-shaped mesenchymal cells (arrowheads), telocyte-like cells (arrows), prospective mesothelium (*), and lumen (Lum). (**D**) Photomicrograph of a sagittal section of a 2-day-old quail embryo showing the stomach primordium (St) is suspended by the dorsal mesentery (Dm) and connected with the liver primordium (Lp) by the dorsal part of the ventral mesentery (Vm). Aorta (Ao), mesonephros (M), and celomatic cavity (*).

The primordium of the stomach could be observed as the most dilated part of the gut, dorsal, and cranial to the liver primordium and ventral to the level of the notochord. It appeared in the form of a spindle-shaped hollow organ with a strongly convex dorsal border and slightly concave ventral border. It continued cranially with the esophagus and caudally with the duodenum (Fig. 1A). Moreover, it was suspended by the dorsal mesentery and the dorsal section of the ventral mesentery extended to the liver primordium. The stomach was lined with a layer of thin endodermal epithelium (Fig. 1D) and a thick mesenchymal layer on the outside. (Fig. 1D).lining and an exterior thick mesenchymal layer. The epithelium appeared to be pseudostratified columnar, with vacuolated basophilic cytoplasm and a vesicular nucleus. The mesenchymal layer was thin cranially (prospective section of the glandular stomach) and thick caudally dorsally (prospective part of muscular stomach). This layer was made up of two types of cells: mesenchymal cells in the shape of stars and telocytes. The nucleus of the star-shaped cells was big and vesicular. Telocytes were spindly-shaped cells with a big oval nucleus and lengthy processes that connected to mesenchymal cells. The mesenchymal layer's outermost layer was darkly stained and consisted of stratified columnar epithelium. This layer represented the tunica serosa’s future mesothelium. Because the mesenchymal part of the wall was greater dorsally, the wall of the stomach primordium was thicker dorsally than ventrally. There were no materials in the lumen (Fig. 1B,C).

### Three-day embryo

**Figure 2:**
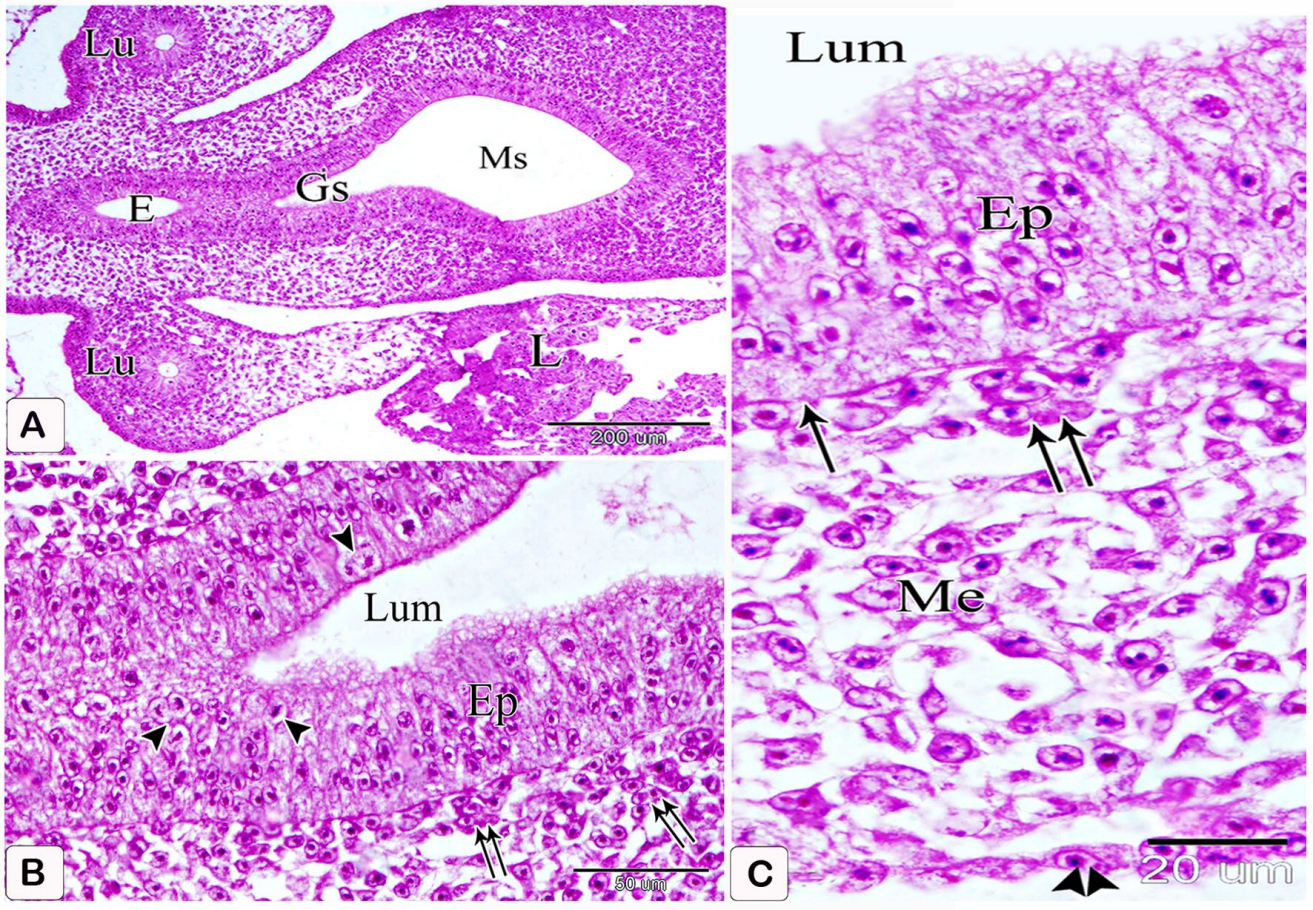
Photomicrographs of paraffin sections in the stomach primordium of a 3-day-old quail embryo stained with hematoxylin and eosin. (**A**) photomicrograph of a frontal section of a 3-day-old quail embryo showing the prospective glandular stomach (Gs) is continued with the esophagus (E) cranially and the prospective muscular stomach (Ms) caudally. Lung (Lu) and liver (L). (**B** and **C**) Photomicrographs of sections in the prospective glandular stomach of a 3-day-old quail embryo showing the structure of its wall. Pseudostratified columnar epithelium (Ep), discontinuous basement membrane (arrows), different stages of mitotic divisions (arrowheads), mesenchyme (Me), mesothelium (double arrowhead), and lumen (Lum). Notice, aggregations of some mesenchymal cells in groups under the epithelium (double arrows).

The stomach could be divided into two distinct parts: a small cranial portion (potential glandular stomach) and a large caudal portion (potential muscular stomach) (Fig. 2A). The pseudostratified columnar epithelium of the glandular stomach contained an ovoid vesicular nucleus. The various phases of mitotic divisions were visible (Fig. 2B). This epithelium was supported by a non-uniform basement membrane. Some mesenchymal cells formed clusters beneath the epithelium. The tunica serosa mesothelium consisted of a single layer of flattened or cuboidal cells with ovoid or rounded nuclei (Fig. 2C).

### Four-day embryo

**Figure 3:**
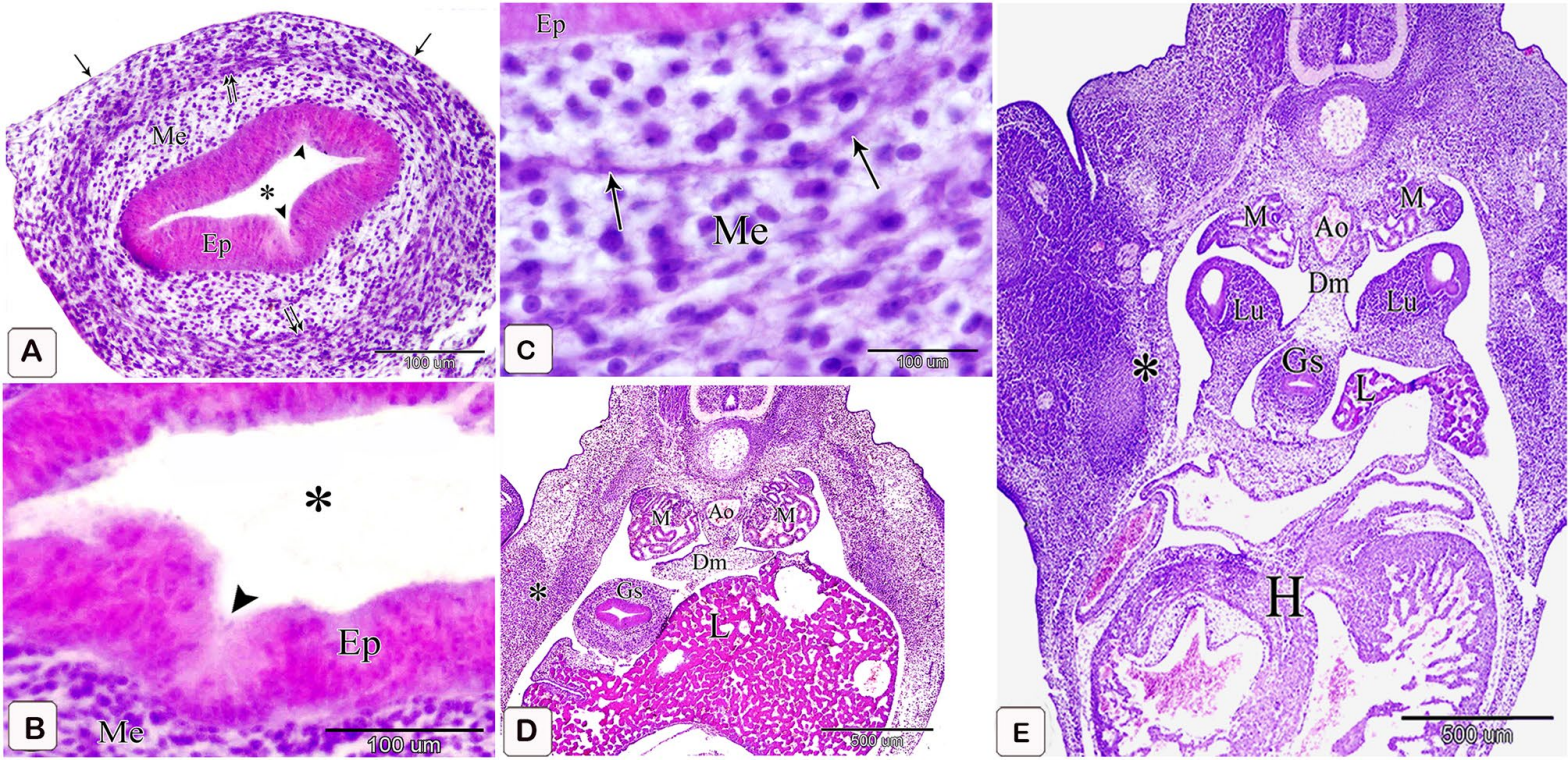
Photomicrographs of paraffin sections in the stomach primordium of a 4-day-old quail embryo stained with hematoxylin and eosin. (**A**,**B**) Photomicrographs of sections in the proventriculus of a 4-day-old quail embryo showing the primordium of the proventricular glands (arrowheads). Epithelium (Ep), mesenchyme (Me), mesothelium (arrows), and lumen (*). Notice the condensation of the mesenchyme (double arrows) nearly in the middle of the mesenchymal layer. (**C**) Photomicrograph of a section in the proventriculus of a 4 day old quail embryo showing differentiation of some mesenchymal cells (Me) into myoblast cells (arrows). Epithelium (Ep). (**D** and **E**) Photomicrographs of transverse sections of a 4 day old quail embryo showing the relations of the glandular stomach (Gs) to the surrounding structures cranially (D) and caudally (E). Liver (L), heart (H), lung (Lu), aorta (Ao), mesonephros (M), and left body wall (*). Notice that the dorsal mesentery (Dm) is longer and oblique caudally.

Evaginations of the lining epithelium marked the appearance of the compound proventricular glands' primordium. The epithelial cells showed shortening and were arranged radially at the base of these evaginations, beneath which the basement membrane evidently slightly protruded into the mesenchyme. Near the middle of the mesenchymal layer, the condensation of the mesenchyme was clearly observed wherein some mesenchymal cells differentiated into myoblast cells forming the prospective smooth muscle layer. Furthermore, the mesothelium became wholly flattened (Fig. 3A–C).

The glandular stomach’s long axis (proventriculus) extended craniocaudally from the median plane to the left side. It was placed just left of the median plane on the cranium, and it was connected medially to the right lobe of the liver, laterally to the left lung, dorsally to the aorta, and ventrally to the heart. Caudally, it was linked ventromedially to the liver, dorsally to the left mesonephros, and laterally to the left body wall. The proventriculus seemed flattened dorsoventrally in the transverse section. The lumen was slit-like and free of materials along its length (Fig. 3D,E).

### Five-day embryo

**Figure 4:**
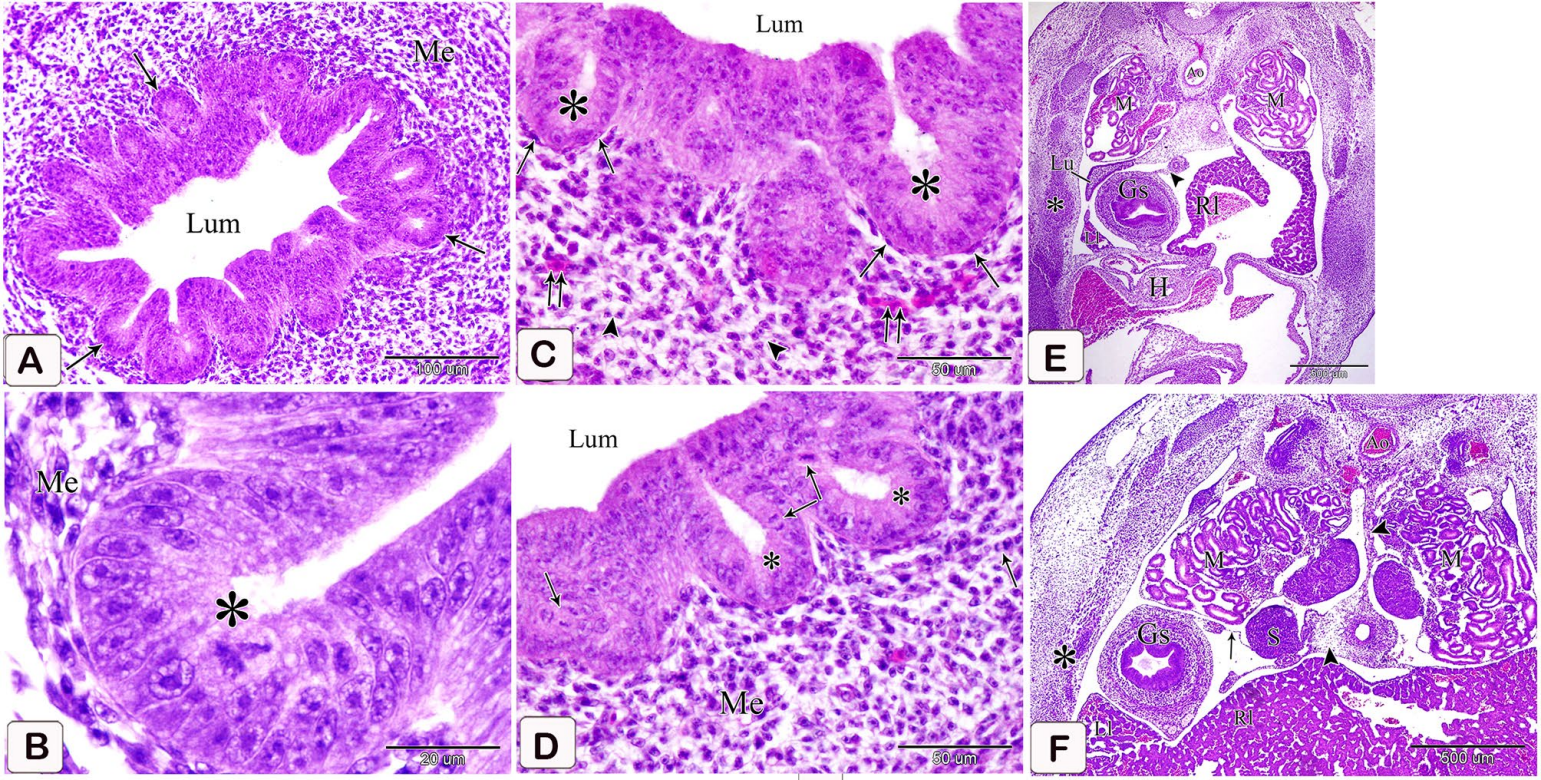
Photomicrographs of paraffin sections in the stomach primordium of a 5-day-old quail embryo stained with hematoxylin and eosin. (**A**) Photomicrograph of a section in the proventriculus of a 5-day-old quail embryo showing the gland primordium (arrows) spreading throughout most of the mucosa. Mesenchyme (Me) and lumen (Lum). (**B**) Photomicrograph of a section in the proventriculus of a 5-day-old quail embryo showing the projection of the gland primordium (*) into the underlying mesenchyme (Me). Notice the special arrangement of the glandular epithelium. (**C**) phomicrograph of a section in the proventriculus of a 5 day old quail embryo showing the condensed, somewhat flattened mesenchymal cells (arrows) under the epithelium of the gland rudiments(*): star-shaped mesenchymal cells (arrowheads) and lumen (Lum). Notice, vascular elements (double arrows) are found within the mesenchymal layer. (**D**) Phomicrograph of a section in the proventriculus of a 5-day-old quail embryo showing mitotic figures (arrows) within the glandular epithelium and the mesenchymal cells. Gland primordium (*), mesenchyme (Me), and lumen (Lum). (**E** and **F**) Photomicrographs of transverse sections of a 5-day-old quail embryo showing the relations of the glandular stomach (Gs) to the surrounding structures cranially (Fig. 18) and caudally (Fig. 19). Right lobe of the liver (Rl), left lobe of the liver (Ll), left lung (Lu), heart (H), aorta (Ao), mesonephros (M), left body wall (*), spleen (S), dorsal mesentery (arrowheads), and gastrosplenic ligament (arrow).

The gland primordium increased in number, spreading throughout most of the mucosa. The special arrangement of the glandular epithelium became clearer and the gland primordium projected more deeply into the underlying mesenchyme. The mesenchymal cells consolidated and flattened under the epithelium of the gland rudiments. Vascular components were found within the mesenchymal layer. Mitotic patterns were found in both the glandular epithelium and the mesenchymal cells (Fig. 4A–D).The cranial part of the glandular stomach became separated laterally from the left body wall by the left lung and left lobe of the liver. Caudally, it was linked to the spleen as well as the liver, and it was linked dorsally to the well-developed mesonephros and ventrally to the liver, giving a deeper gastric impression. The dorsal mesentery extended obliquely from the roof of the body cavity ventrally and to the left to be attached to the dorsal aspect of the glandular stomach. It increased in length caudal wards and extended vertically and then transversely. Its caudal attachment with the proventriculus was interrupted by the spleen forming the gastrosplenic ligament (Fig. 4E,F).

### Six-day embryo

**Figure 5:**
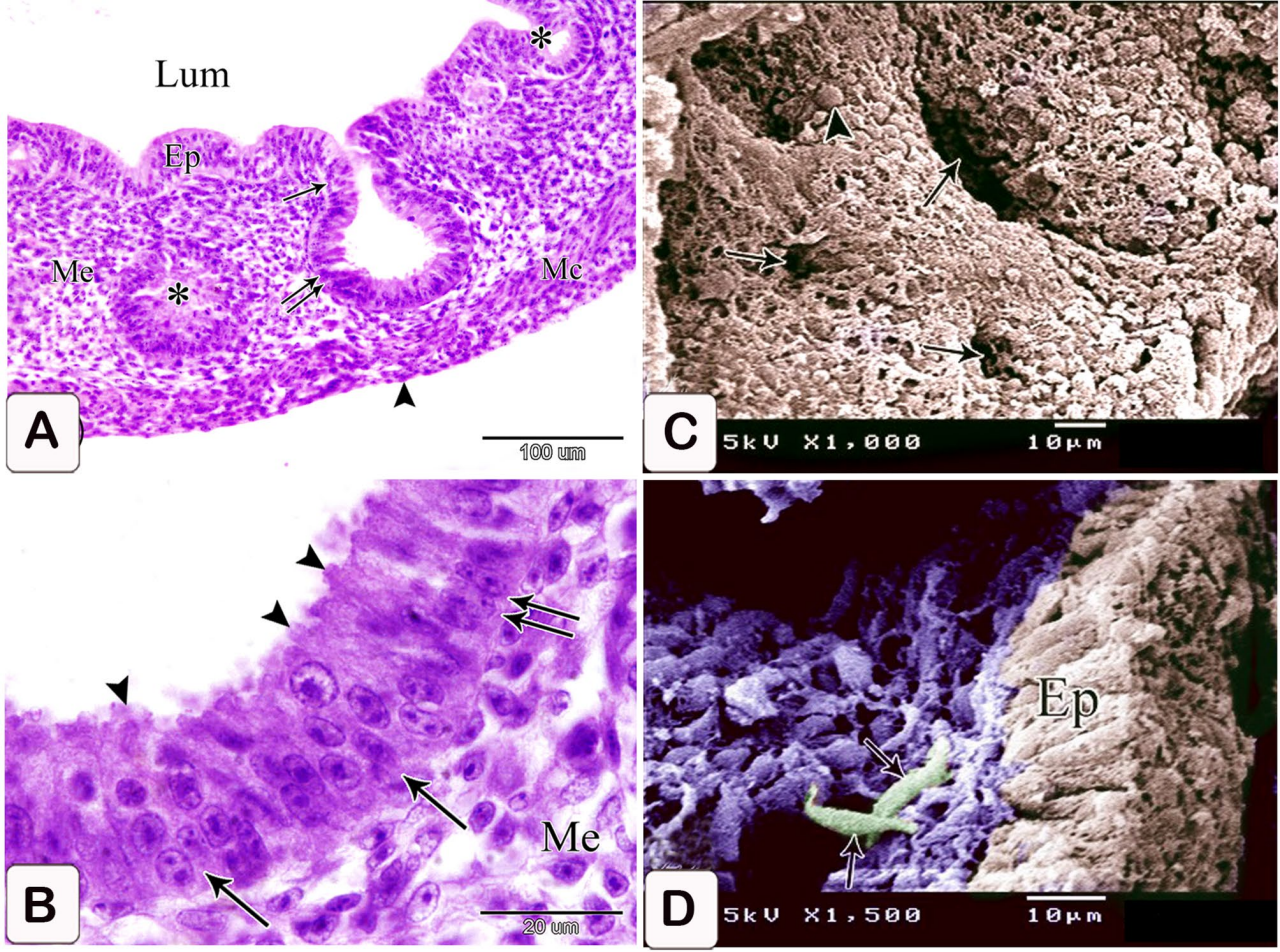
Photomicrographs of a digital coloring scanning electron micrograph and paraffin sections stained with hematoxylin and eosin of the stomach primordium of a 6-day-old quail embryo. (**A**) Phomicrograph of a paraffin section stained with hematoxylin and eosin in the proventriculus of a 6-day-old quail embryo showing varying degrees of development in the proventricular glands (*). Lumen (Lum), luminal epithelium (Ep), mesenchyme (Me), muscular coat (Mc), and mesothelium (arrowhead). Notice, some proventricular glands consist of a spherical tip (double arrow) and a stalk (arrow). (**B**) Phomicrographs of paraffin sections stained with hematoxylin and eosin in the proventriculus of a 6 day old quail embryo show the glandular epithelium is pseudostratified columnar in some areas (arrow) and simple columnar in others (double arrow). Notice that theapical portion of most of the glandular epithelium is elongated and slender (arrowheads), giving it a serrated appearance. Mesenchyme (Me). (**C**) Scanning electron micrographs of the luminal surface of the proventriculus of a 6-day-old quail embryo show that the on the surface of the proventricular epithelial cells (arrowheads) is polygonal in shape. Notice thon the openings of the gland rudiments (arrows) appear as hollows of variable shapes and sizes on the luminal surface. (**D**) Scanning electron micrograph of the proventriculus of a 6-day-old quail embryo showing telocytes (arrows) under the epithelium (Ep).

The proventricular glands showed varying degrees of development. Some of them became more developed, protruding deeply into the underlying mesenchyme. They took the simple alveolar profile consisting of a spherical tip and a stalk representing the future primary duct. The gland epithelium was pseudostratified columnar in some regions and simple columnar in others. Most glandular epithelium’s apical section became elongated, and thin, and protruded into the lumen beyond the point of cell connections, giving the epithelium a serrated look. (Fig. 5A,B).

Scanning electron microscopy showed that the proventricular epithelial cells had a polygonal form on their surface. On the surface of the lumen, the apertures of the gland rudiments were seen as hollows of different sizes and shapes (Fig. 5C). Telocytes with several processes were observed under the epithelium (Fig. 5D).

### Seven-day embryo

**Figure 6:**
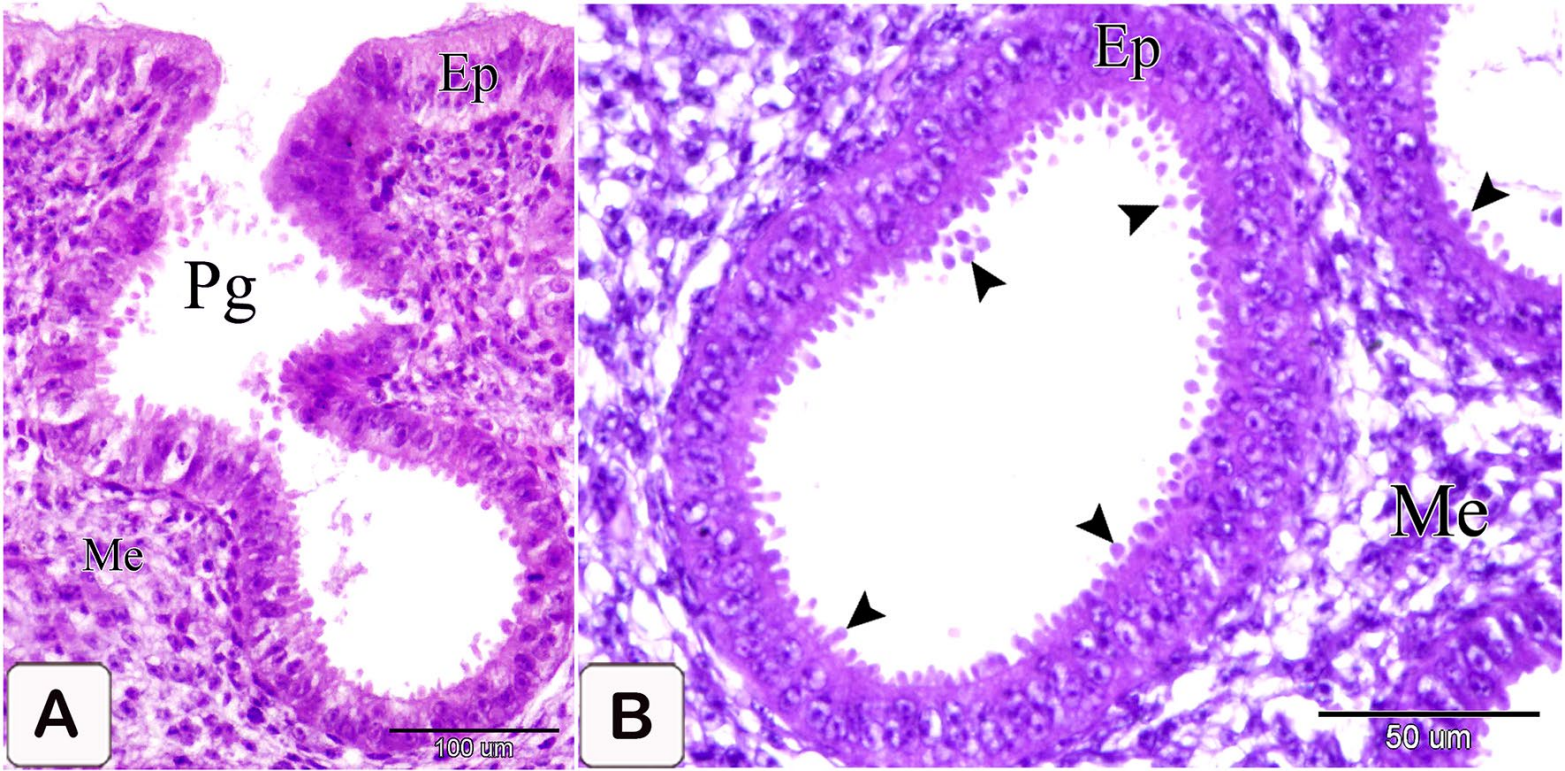
Photomicrographs of paraffin sections stained with hematoxylin and eosin in the stomach primordium of a 7-day-old quail embryo. (**A**) Photomicrograph of a semi thin section stained with toluidine blue in the proventriculus of a 7-day-old quail embryo showing elongation of the proventricular gland (Pg) without branching. Epithelium (Ep) and mesenchyme (Me). (**B**) Photomicrograph of a semithin section stained with toluidine blue in the proventriculus of a 7 day old quail embryo showing profuse finger-like projections of the glandular epithelium (Ep) bulging into the lumen and some of them sloughing off (arrowheads). Mesenchyme (Me).

**Figure 7:**
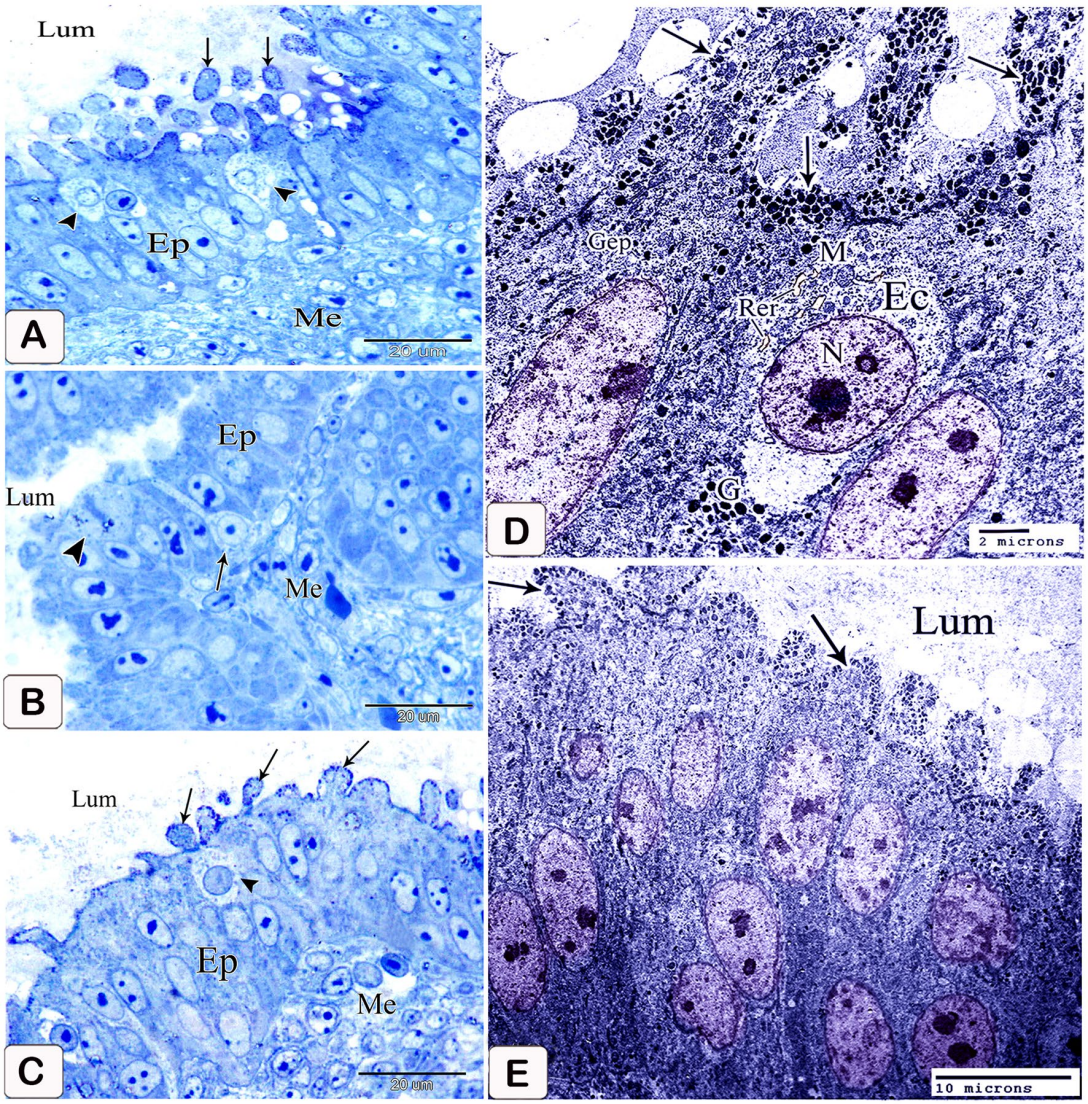
Photomicrographs of a semi-thin section stained with toluidine blue and a digital coloring transmission electron micrograph in the stomach primordium of a 7-day-old quail embryo. (**A**) Photomicrograph of a semi-thin section stained with toluidine blue in the proventriculus of a 7-day-old quail embryo showing the apical end of the glandular epithelium (EP) as well as the projections containing intense blue granules (arrows). Notice the open-type endocrine cells (arrowheads) present in between the glandular epithelium. Lumen (Lum) and mesenchyme (Me). (**B**) Photomicrograph of a semi-thin section in the proventriculus of a 7-day-old quail embryo showing some of the open-type endocrine cells (arrow) have narrow elongated cytoplasmic processes towards the luminal surface and others show mitotic figures (arrowhead). Glandular epithelium (EP), lumen (Lum), and mesenchyme (Me). (**C**) photomicrograph of a semi-thin section in the proventriculus of a 7-day-old quail embryo shows the projections on the apical portion of the luminal epithelium (EP) are few and small. Notice, the apical end of the luminal epithelium and its projections contain intense blue granules (arrows). Open-type endocrine cells (arrowhead), lumen (Lum), and mesenchyme (Me). (**D**) Transmission electron micrograph of the proventriculus of a 7-day-old quail embryo showing the ultrastructure of the open-type endocrine cell (Ec). Mitochondria (M), rough endoplasmic reticulum (Rer), electron-dense granules (G), and nucleus (N). Notice, the glandular epithelium (Gep) and its projections contain numerous electron-dense granules of different sizes and shapes in their apical border (arrows). (**E**) A transmission electron micrograph of the proventriculus of a 7-day-old quail embryo shows the pseudostratified columnar epithelium lining the lumen (Lum) and its projections, which have many electron-dense granules at their tips (arrows).

The glands elongated without branching. The elongation of the apical portion of the glandular epithelium became evident forming profuse finger-like projections bulged into the lumen and some of them sloughed off. These projections were observed on the apical portion of the luminal epithelium, but they were fewer and smaller. Semithin sections showed that granules stained with toluidine blue were at the end of the luminal and glandular epithelium as well as the projections. Moreover, pale oval cells with rounded nuclei were observed in between the luminal and glandular epithelium. Some of them possessed a narrow elongated cytoplasmic process pointing toward the luminal surface and a long axis perpendicular to thebasement membrane. Moreover, some cells showed mitotic figures. These cells were suggested to be the open-type endocrine cells with luminal contact (Figs. 6A,B and 7A–C).

The luminal epithelium was pseudostratified columnar in type, with rounded or oval nuclei having 1–3 nucleoli, as revealed by transmission electron microscopy pictures. Numerous electron-dense granules of various sizes and shapes were seen in the luminal and glandular epithelium, as well as the projections. Open-type endocrine cells were detected in the glandular epithelium. They showed an electron-lucent cytoplasm with supranuclear mitochondria, rough endoplasmic reticulum, and Golgi apparatus. Few pleomorphic electron-dense granules were observed in a basal location. The nucleus was rounded with a prominent nucleolus, distinct nuclear membrane, and marked nuclear pores (Fig. 7D,E).

**Figure 8:**
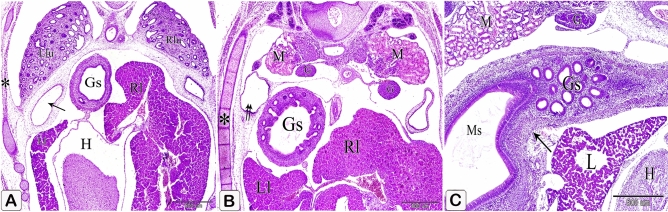
Photomicrographs of paraffin sections stained with hematoxylin and eosin in the stomach primordium of a 7-day-old quail embryo. (**A**,**B**) Photomicrographs of transverse sections of a 7-day-old quail embryo showing the glandular stomach (Gs) is separated from the body wall (*) cranially by the left lung (Llu) as well as the cranial thoracic air sac (arrow) and caudally by the left abdominal air sac (double arrow). Right lobe of the liver (Rl), left lobe of the liver (Ll), heart (H), right lung (Rlu), mesonephros (M), and gonad (G). (**A**) is a cranial level, and (**B**) is a caudal level. (**C**) Photomicrograph of a sagittal section of a 7-day-old quail embryo showing the glandular stomach (Gs) is spindle shaped in outline and a slight indentation (arrow) demarcating it from the muscular stomach (Ms) ventrally. Heart (H), liver (L), mesonephros (M), and gonad (G).

The glandular stomach was positioned in the left dorsal quadrant of the body cavity. It became separated from the left body wall cranially (by the left lung) as well as from the left cranial thoracic air sac caudally (by the left abdominal air sac). Caudally, it was related dorsally to the gonad along with the mesonephros (Fig. 8A,B). Along its longitudinal axis, the glandular stomach was spindle-shaped in outline with its broad end located caudally and the narrow end located cranially. A slight constriction could be observed cranially demarcating it from the esophagus. Caudally, a slight indentation could be detected between the glandular stomach and the muscular stomach ventrally, whereas dorsally, they continued without any line of demarcation (Fig. 8C).

### Eight-day embryo

**Figure 9:**
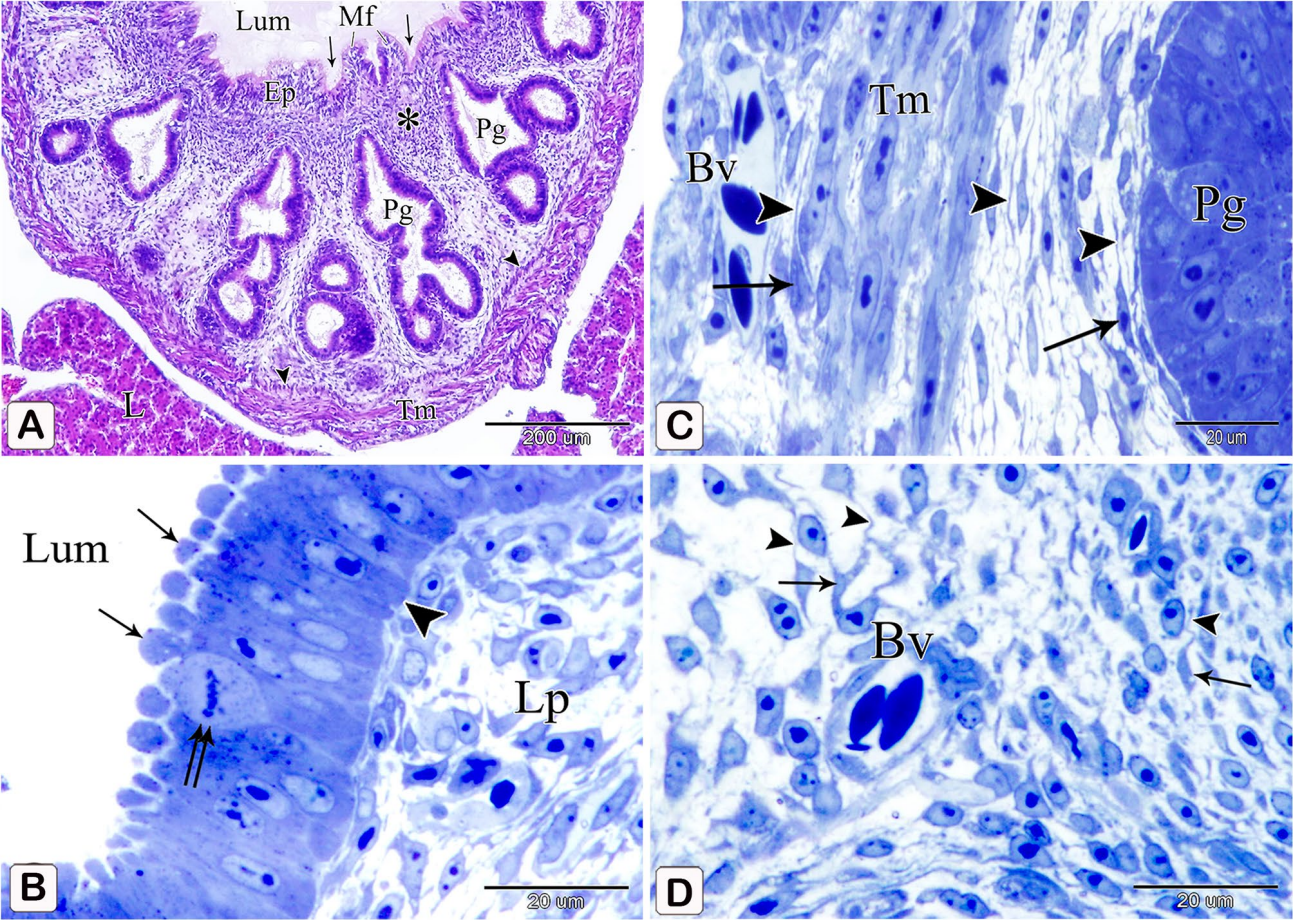
Photomicrographs of paraffin sections stained with hematoxylin and eosin and semi-thin sections stained with toluidine blue in the stomach primordium of an 8-day-old quail embryo. (**A**) Photomicrograph of a paraffin transverse section of an 8-day-old quail embryo showing the proventricular mucosal surface exhibits many small folds (Mf) with intervening depressions (arrows). Notice, condensation of the mesenchyme (*) under the luminal epithelium (Ep) and the branching of the proventricular glands (Pg). Lumen (Lum), outer layer of muscularis mucosae (arrowheads), tunica muscularis (Tm), and liver (L). (**B**) Photomicrograph of a semi-thin section in the proventriculus of an 8 day old quail embryo showing the projections of the glandular epithelium (arrows) are more prominent. Notice that most of the glandular epithelium is simple columnar (arrowhead). Open-type endocrine cells (double arrow), lumen (Lum), and lamina propria (Lp). (**C** and **D**) Photomicrographs of semi thin sections in the proventriculus of an 8-day-old quail embryo showing telocytes (arrows) with long telopodes (arrowheads) around the proventricular glands (Pg), muscles (Tm), and blood vessels (Bv).

The proventricular mucosal surface showed many small folds (plicae) with intervening depressions (sulci). The proventricular glands began to be branched. Most of the glandular epithelium became simple columnar in type, whereas the pseudostratified columnar epithelium was observed in small areas. The apical projections became increasingly apparent, and the lumina of the glands revealed a significant amount of cellular debris containing nuclei. Open-type endocrine cells were observed in between the luminal and glandular epithelium. Some of these cells had narrow elongated cytoplasmic process toward the basement membrane (Fig. 9A,B). Telocytes with long processes (telopodes) were observed in the mesenchyme around the glands, muscles, and blood vessels (Fig. 9C,D).

**Figure 10:**
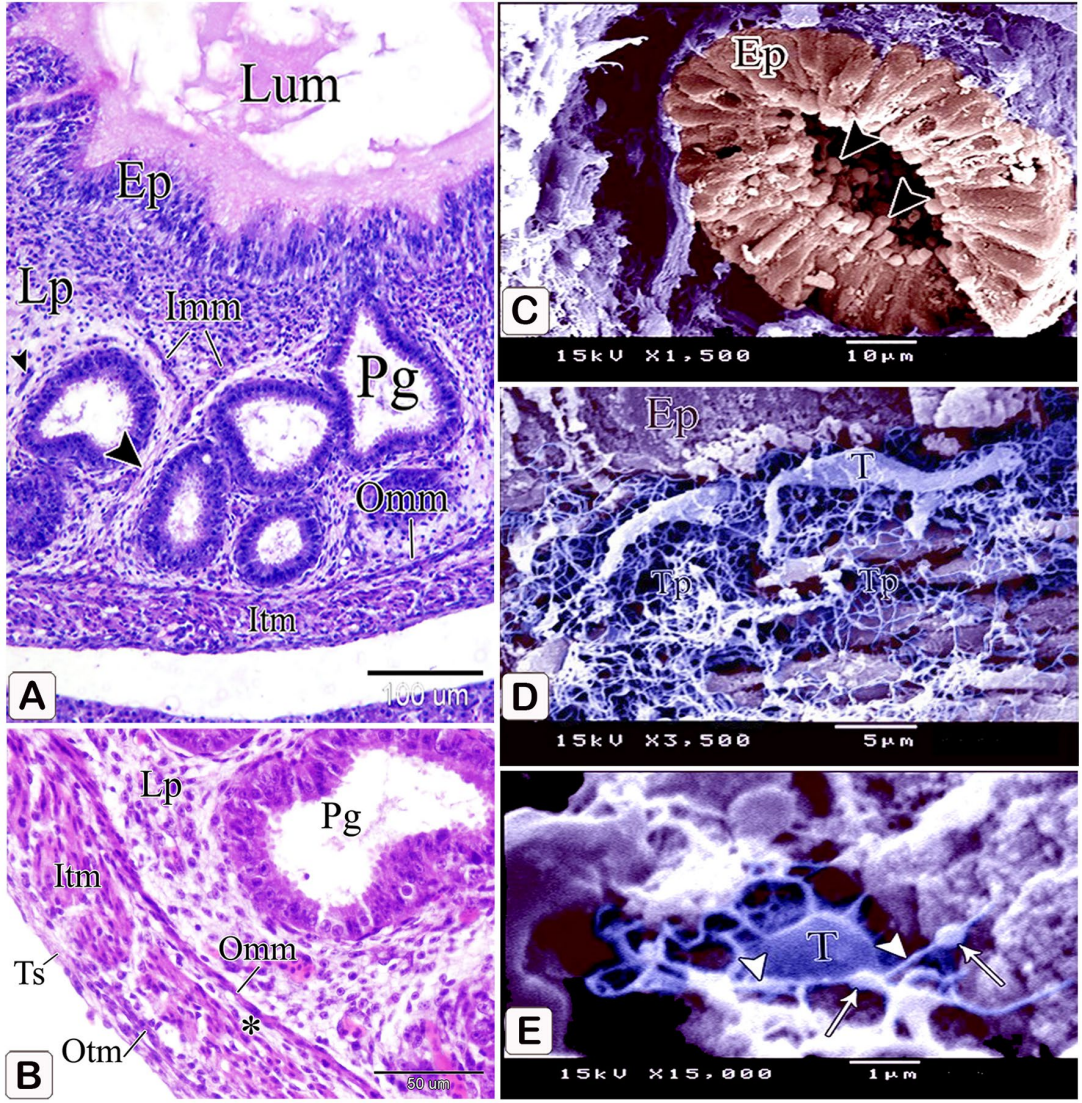
Photomicrographs of paraffin sections stained with hematoxylin and digital coloring of scanning electron micrographs in the stomach primordium of an 8-day-old quail embryo. (**A**,**B**) Photomicrographs of sections in the proventriculus of an 8-day-old quail embryo showing differentiation of the muscular coat. Lumen (Lum), luminal epithelium (Ep), proventricular glands (Pg), lamina propria (Lp), inner muscularis mucosae (Imm), outer muscularis mucosae (Omm), inner tunica muscularis (Itm), outer tunica muscularis (Otm), tunica submucosa (*) and tunica serosa (Ts). Notice that a few muscle fibers (arrowheads) extend between the glands. (**C**) digital coloring of a scanning electron micrograph of the proventriculus of an 8-day-old quail embryo showing very prominent finger-like projections (arrowheads) on the apical portion of the glandular epithelium (Ep). (**D**,**E**) digital coloring of a scanning electron micrograph of the proventriculus of an 8-day-old quail embryo showing a network of telocytes (T) under the luminal epithelium (Ep). Notice the telopodes (Tp) consist of alternating podoms (arrows) and podomeres (arrowheads), giving it a beading appearance.

The muscular coat was differentiated into lamina muscularis mucosae and tunica muscularis. The lamina muscularis mucosae consisted of a thin discontinuous inner layer and continuous outer layer with few muscle fibers extending between the glands. The tunica muscularis had two layers as well: a thick inner circular layer and a thin discontinuous outer longitudinal layer. The tunica submucosa, which lay between the outer layer of muscularis mucosae and the inner layer of tunica muscularis, was highly thin. (Fig. 10A,B).

The scanning electron microscopic images confirmed the light microscopic results wherein most of the glandular epithelium became simple columnar in type with very prominent finger-like projections on its apical portion (Fig. 10C). A network of telocytes was observed under the luminal epithelium. The telocytes were connected with each other and with the surrounding structures. The telopodes consisted of alternating thin segments (podomeres) and dilations (podoms), giving it a beading appearance (Fig. 10D,E).

### Nine-day embryo

**Figure 11:**
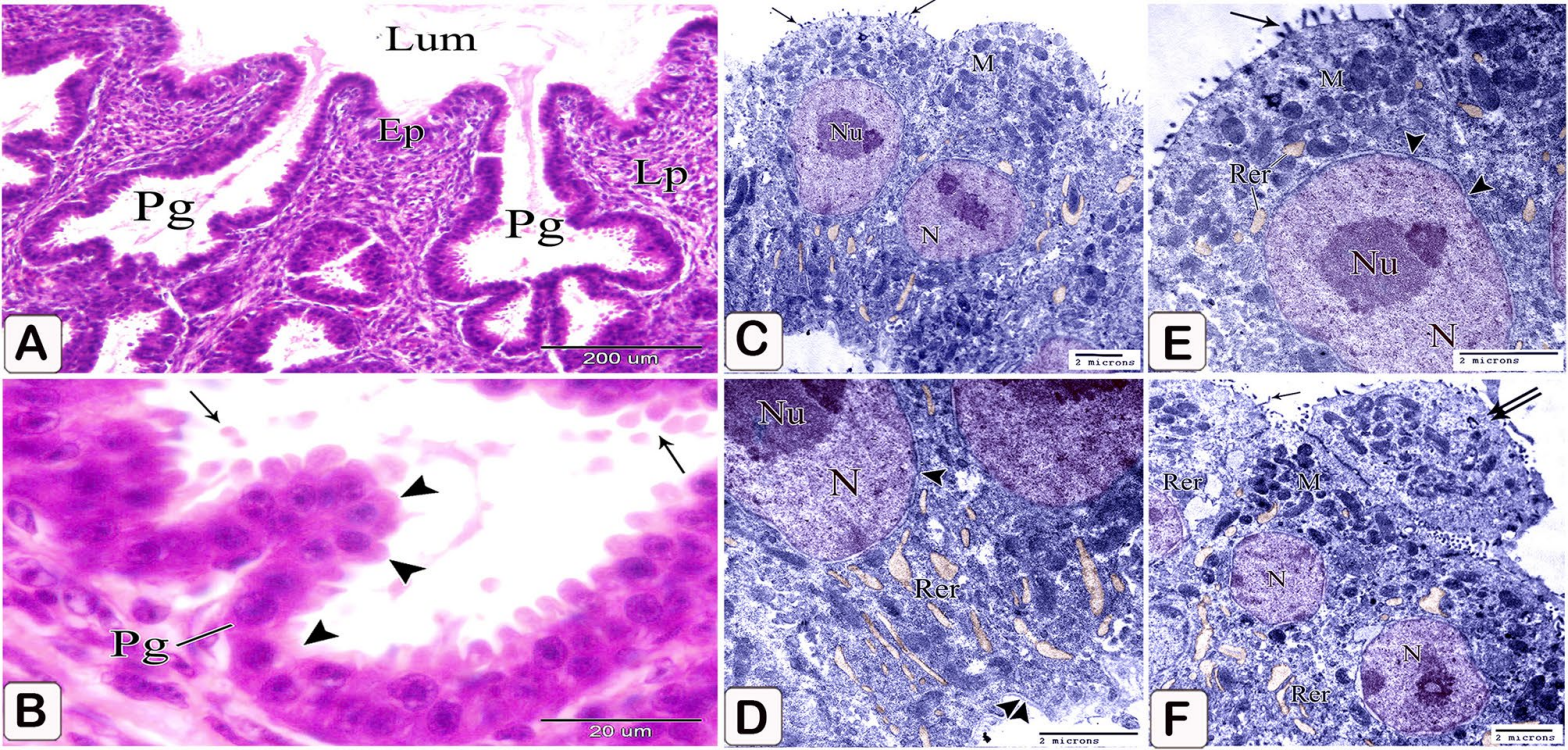
Photomicrographs of paraffin sections stained with hematoxylin and digital coloring of transmission electron micrographs in the stomach primordium of a 9-day-old quail embryo. (**A,B**) Photomicrograph of a section in the proventriculus of a 9-day-old quail embryo showing transformation of some of the epithelial cells lining the proventricular glands (Pg) into low columnar cells (arrowheads). Notice the sloughing of the apical portion of the glandular epithelium (arrows). (**C**–**E**) digital coloring of transmission electron micrographs of the proventriculus of a 9 day old quail embryo showing the ultrastructure of the low columnar cells lining the proventricular glands. Microvillus-like structures (arrows), mitochondria (M), rough endoplasmic reticulum (Rer), nucleus (N), nucleolus (Nu), nuclear pores (arrowheads), and basement membrane (double arrowhead). Notice that at apical portion of some cells projects into the lumen to be sloughed off (double arrow).

Branching of the proventricular glands increased (Fig. 11A). Sloughing of the apical portion of the glandular epithelium became greater, and some of the epithelial cells lining the glands as well as the ducts transformed into low columnar cells (Fig. 11B). Ultra-structurally, the low columnar cells lining the glands showed some short microvillus-like structures on their apical border. The cytoplasm contained numerous mitochondria mainly at the apical portion of the cell and abundant rough endoplasmic reticulum concentrated at its basal portion. A large number of free ribosomes are distributed throughout the cytoplasm. Some cells' apical portions protruded into the lumen to be sloughed off. The centrally located nuclei were rounded or oval in shape with evenly distributed chromatin and distinct nucleolus. Moreover, the nuclear membrane was distinct with marked nuclear pores (Fig. 11C–F).

**Figure 12:**
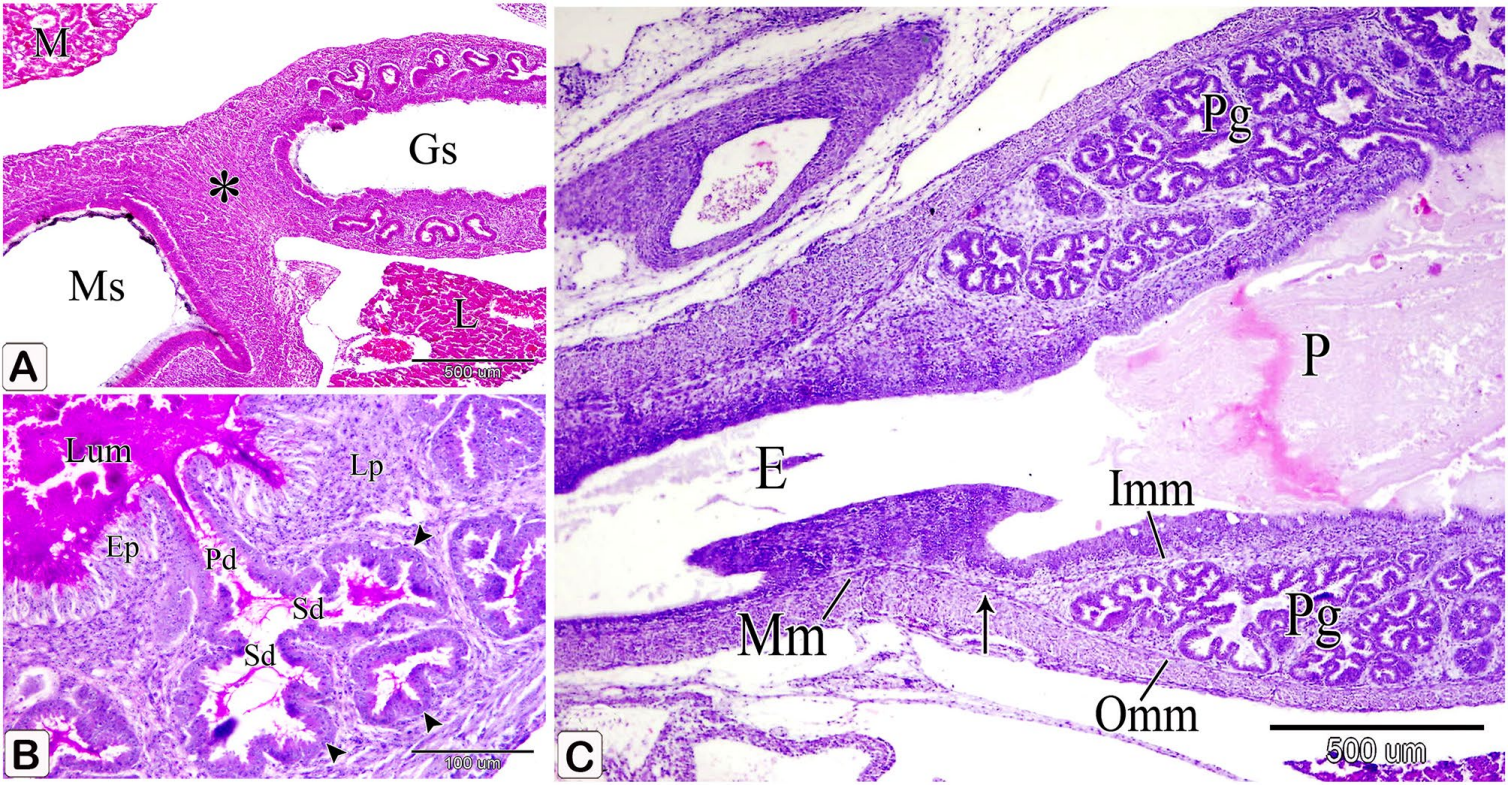
Photomicrographs of a paraffin section of a 9-day-old quail embryo and a 10-day-old quail embryo. (**A**) photomicrograph of a sagittal section of a 9-day-old quail embryo showing the isthmus (*) between the glandular stomach (Gs) and the muscular stomach (Ms). Mesonephros (M) and liver (L). (H&E). (**B**) Photomicrograph of a section in the proventriculus of a 10-day-old quail embryo showing division of the primary duct (Pd) of the proventricular gland into two secondary ducts (Sd). Secretory units (arrowheads), lumen (Lum), luminal epithelium (Ep), and lamina propria (Lp). (PAS). (**C**) photomicrograph of a sagittal section of a 10 day old quail embryo showing the muscularis mucosae (Mm) of the esophagus (E) diverges at the esophago-proventricular junction (arrow) into inner and outer layers, which continue with the inner (Imm) and outer (Omm) layers of the muscularis mucosae of the proventriculus (P). Proventricular glands (Pg) (H&E).

A slight indentation appeared between the glandular stomach and the muscular stomach dorsally, whereas the ventral indentation became deeper forming the isthmus (Fig. 12A).

### Ten-day embryo

The primary duct of the proventricular gland is divided into two secondary ducts connecting it with the secretory units (Fig. 12B). The muscularis mucosae of the esophagus are divided into inner and outer layers at the esophago-proventricular junction, which continued with the inner and outer layers of the muscularis mucosae of the proventriculus. The outer layer was thick, whereas the inner layer appeared thin and discontinuous, enclosing the compound proventricular glands in between (Fig. 12C).

### Eleven-day embryo

**Figure 13:**
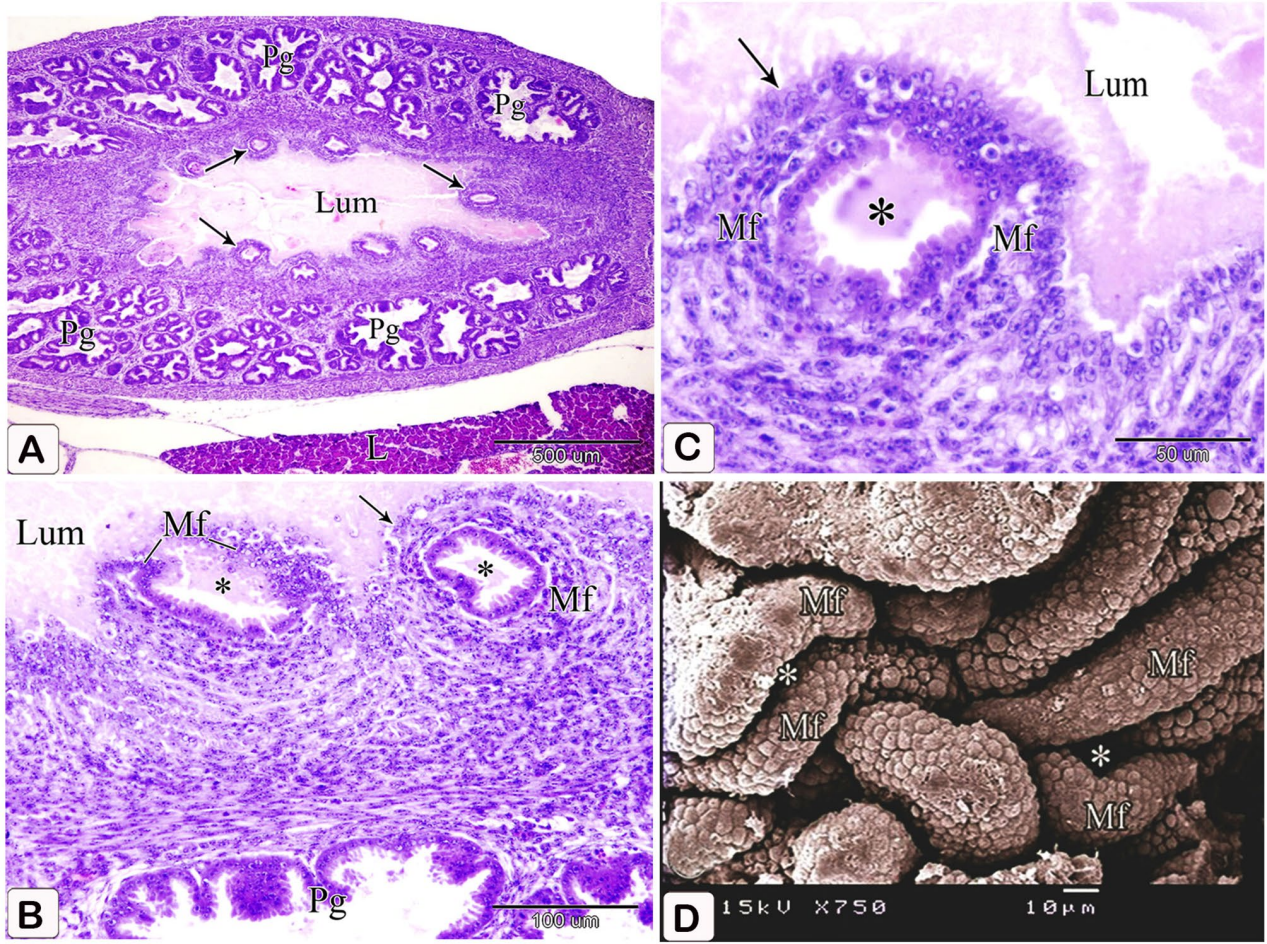
Photomicrographs of a paraffin section of an 11-day-old quail embryo stained with hematoxylin and eosin and digital coloring of a transmission electron micrograph of a 10-day-old quail embryo. (**A**–**C**) Photomicrographs of paraffin sagittal sections of an 11-day-old quail embryo showing several papillae on the mucosal surface of the proventriculus (arrows). Mucosal folds (Mf), openings (*) of proventricular glands (Pg), lumen (Lum), and liver (L). (**D**) digital coloring of A scanning electron micrograph of the luminal surface of the proventriculus of an 11-day-old quail embryo shows the mucosal folds (Mf) are well developed and longer with deep intervening sulci (*).

The mucosal folds became more developed and the adjacent folds approached each other and connected to surround the openings of the proventricular glands forming the mucosal papillae (Fig. 13A–D).

**Figure 14:**
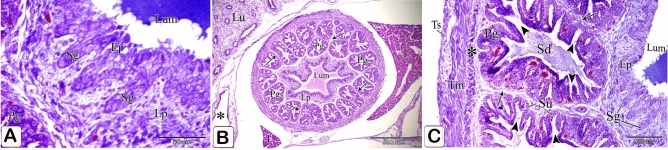
Photomicrographs of a paraffin section of an 11-day-old quail embryo. (**A**) Photomicrograph of a paraffin section in the proventriculus of an 11-day-old quail embryo showing the primordium of the simple tubular glands (Sg) as localized down-growths of the luminal epithelium (Ep) into the lamina propria (Lp) forming solid cords. Lumen (Lum) and proventricular glands (Pg). (Mallory's trichrome stain). (**B**) Photomicrograph of a paraffin transverse section of an 11-day-old quail embryo showing numerous rounded or polymorphic lobules of the proventricular glands (Pg) within the lamina propria (Lp) forming the thickest portion of the wall. Notice that the on thelobules are arranged in small groups separated by connective tissue septa (arrows). Lumen (Lum), lung (Lu), cranial thoracic air sac (*), and liver (L) (H&E). (**C**) Photomicrograph of a paraffin asection in the proventriculus of an 11-day-old quail embryo showing the proventricular glands (Pg) as compound tubuloalveolar glands where the secretory units (Su) are connected to the secondary ducts (Sd) by tertiary ducts (arrowheads). Lumen (Lum), luminal epithelium (Ep), primordium of the simple tubular glands (Sg), connective tissue septa (arrows), outer muscularis mucosae (*), tunica muscularis (Tm), and tunica serosa (Ts) (Mallory’s trichrome stain).

The primordium of the simple tubular glands could be detected as localized down-growths of the luminal epithelial cells into the lamina propria forming solid cords (Fig. 14A).

The proventricular glands established themselves as compound tubuloalveolar glands within the lamina propria, constituting the thickest portion of the proventricular wall. These glands consisted of numerous round or polymorphic lobules arranged in small groups and separated by septa of connective tissue. The septa were composed mainly of collagenous fibers. The secretory units connected to the secondary ducts by tertiary ducts, which were lined by simple cuboidal epithelium (Fig. 14B,C).

**Figure 15:**
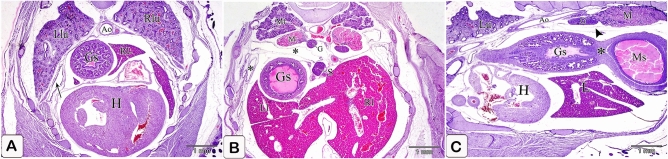
Photomicrographs of paraffin sections of an 11-day-old quail embryo. (**A**,**B**) Photomicrographs of transverse sections of an 11-day-old quail embryo showing the relations of the glandular stomach (Gs) to the surrounding structures cranially (fig.A) and caudally (figure B). Right lobe of the liver (Rl), left lobe of the liver (Ll), heart (H), right lung (Rlu), left lung (Llu), aorta (Ao), metanephros (Mt), spleen (S), and cranial thoracic air sac (arrow). Notice that the glandular stomach is separated from the mesonephros (M) and gonad (G) by the left abdominal air sac (*). (H&E). (**C**) Photomicrograph of a sagittal section of an 11-day-old quail embryo shows the isthmus (*) is well established between the glandular stomach (Gs) and the muscular stomach (Ms). Heart (H), liver (L), lung (Lu), aorta (Ao), gonad (G), mesonephros (M), and the left abdominal air sac (arrowhead). (H&E).

Cranially, the relations of the glandular stomach were similar to the previous stages, but caudally, it was separated from the mesonephros and gonad by the dorsally extended left abdominal air sac (Fig. 15A,B). The dorsal indentation between the glandular stomach and the muscular stomach became deeper and the isthmus became well-defined (Fig. 15C).

### Twelve-day embryo

**Figure 16:**
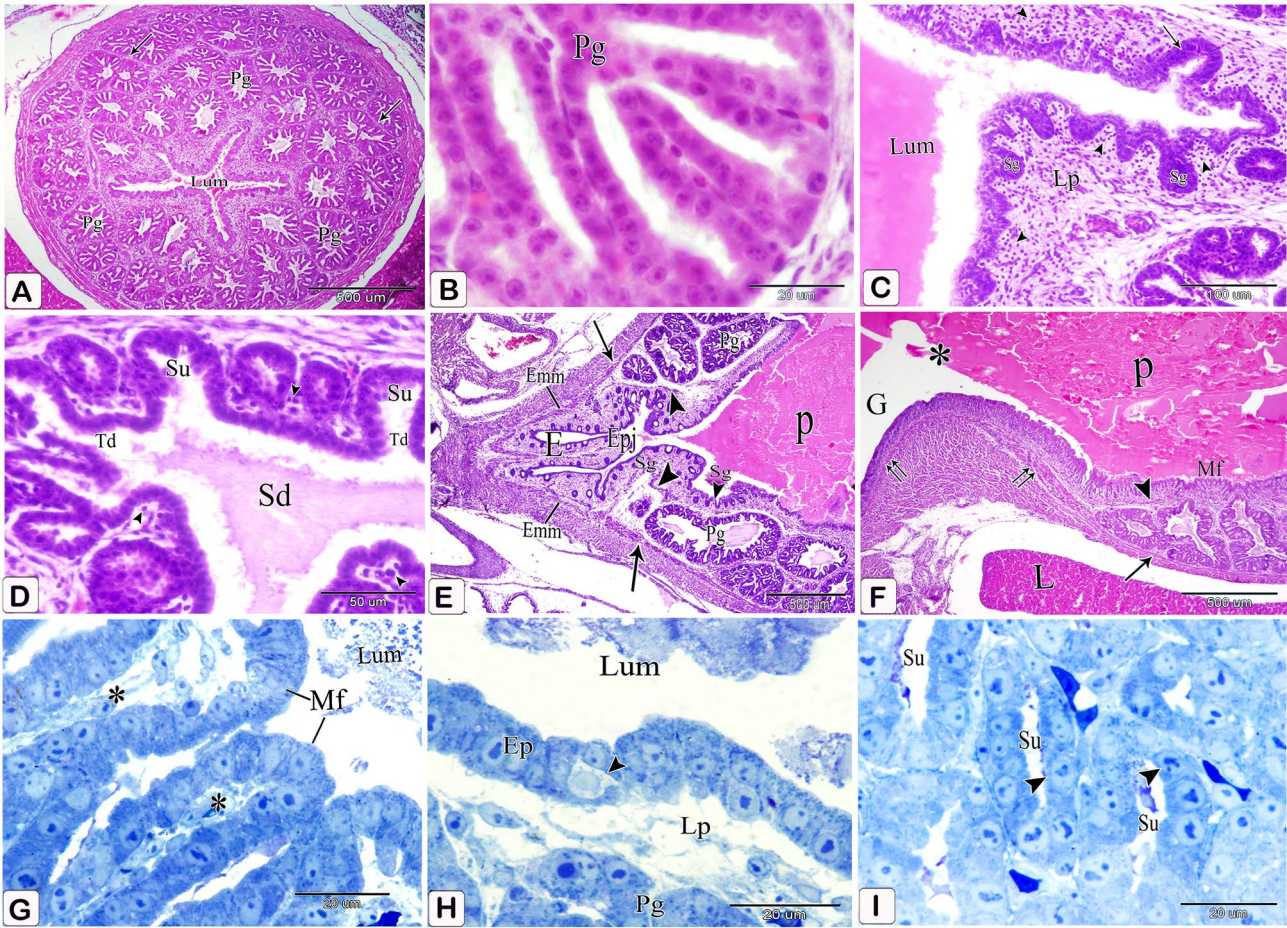
Photomicrographs of paraffin and semi-thin sections of a 12-day-old quail embryo. (**A**,**B**) Photomicrographs of sections in the proventriculus of a 12-day-old quail embryo showing the compound tubuloalveolar glands (Pg) are more branched and most of their lining epithelium is of low columnar type. Notice that the connective tissue septa (arrows) separating the gland lobules became thinner. Lumen (Lum). (H&E). (**C**,**D**) Photomicrographs of sections in the proventriculus of a 12-day-old quail embryo showing the lamina propria (Lp), under the luminal epithelium, and around the compound glands (Pg). Notice the increased number of simple tubular glands (Sg) and that very few of them begin to be canalized (arrow). Lumen (Lum), secretory units (Su), secondary ducts (Sd), and tertiary ducts (Td). (H&E). (**E**,**F**) Photomicrographs of sagittal sections of a 13-day-old quail embryo showing the muscularis mucosae of the esophagus (Emm) diverge at the esophago-proventricular junction (Epj) into inner and outer layers that continue with the inner (arrowheads) and outer (arrows) layers of the muscularis mucosae of the proventriculus (P), which re-join prior to the isthmus (*) to continue with the muscularis mucosae of the gizzard (double arrows). Proventricular glands (Pg), gizzard (G), and liver (L) (H&E). (**G**) Photomicrograph of a semithin section in the proventriculus of a 13 day old quail embryo showing the mucosal folds (Mf) are longer and have a connective tissue core of lamina propria (*). Lumen (Lum). (Toluidine blue). (**H**) Photomicrograph of a semithin section in the proventriculus of a 13-day-old quail embryo showing the closed-type endocrine cells (arrowhead) in between the luminal epithelium (Ep). Lumen (Lum), lamina properia (Lp), and proventricular glands (Pg) (Toluidine blue). (**I**) Photomicrographs of sections in the proventriculus of a 13-day-old quail embryo showing the lining epithelium of the secretory units (Su) of the compound glands consist of low columnar cells with some cuboidal cells (arrowheads) in between.

The compound tubuloalveolar glands became more branched and most of their lining epithelium was of low columnar type. The connective tissue septa separating the gland lobules became thinner (Figs. 16A,B).

The simple tubular glands increased in number and very few of them began to be canalized (Fig. 16C).

### Thirteen-day embryo

Some simple tubular glands became more canalized (Fig. 16D). The inner and outer layers of the muscularis mucosae re-joined prior to the isthmus (Fig. 16E,F). The mucosal folds became longer, lined with simple columnar epithelium, and had a connective tissue core of lamina propria (Fig. 16G). Closed-type endocrine cells were observed in the luminal epithelium. These cells had no luminal contact. They were pale, with rounded nuclei, and possess short bipolar cytoplasmic processes (Fig. 16H). The lining epithelium of the compound glands’ secretory units was composed of low columnar cells with few cuboidal cells interspersed (Fig. 16I).

### Fifteen-day embryo

**Figure 17:**
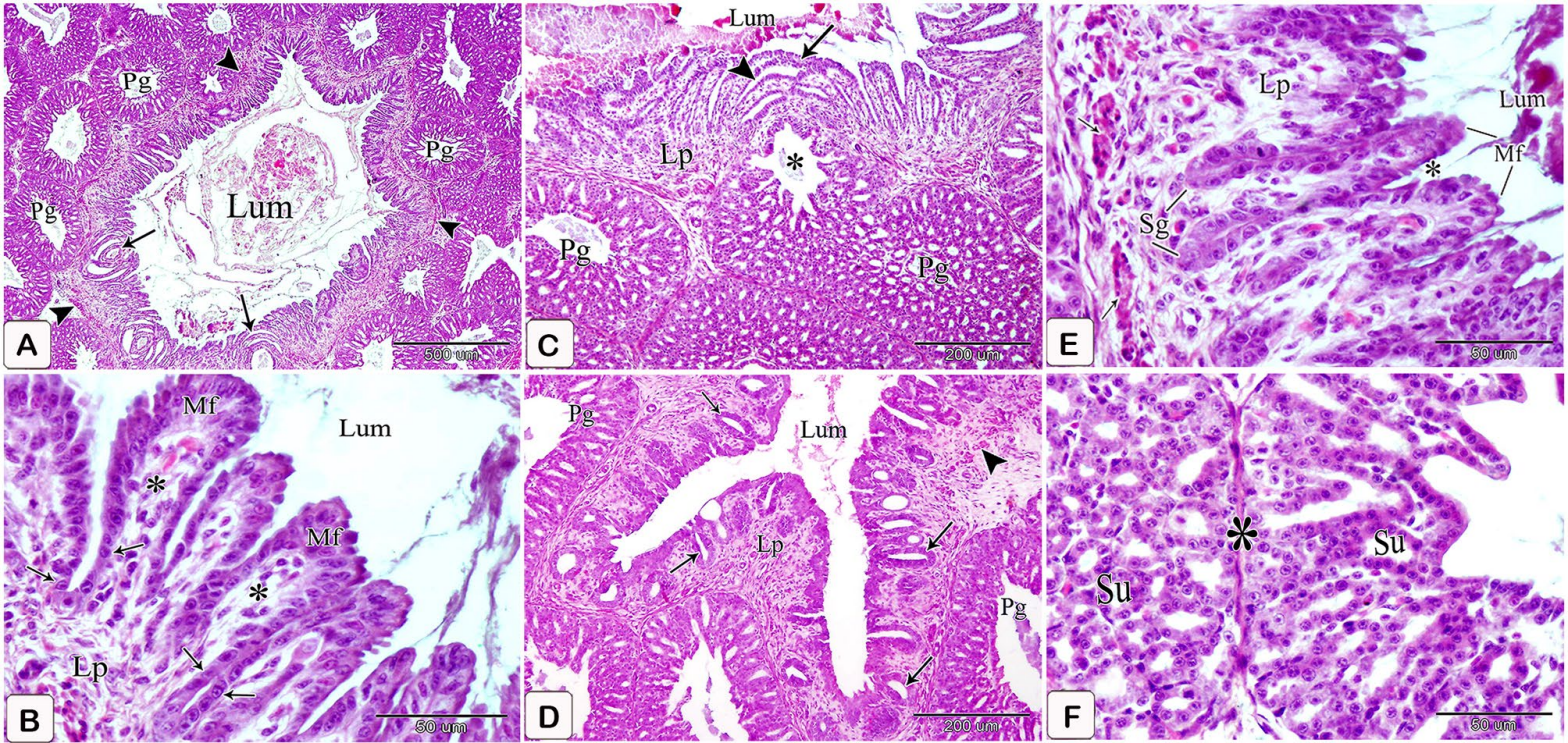
Photomicrographs of paraffin and semi-thin sections of a 15-day-old quail embryo. (**A**) Photomicrograph of a section in the proventriculus of a 15-day-old quail embryo showing the spiral appearance of some mucosal papillae (arrows). Lumen (Lum), inner layer of muscularis mucosae (arrowhead), and proventricular glands (Pg). (H & E). (**B**) Photomicrograph of a section in the proventriculus of a 15 day old quail embryo showing additional folds (arrow) and sulci (arrowhead) are arranged concentrically around a single glandular duct opening (*), giving the mucosal papilla a spiral appearance. Lumen (Lum), lamina properia (Lp), and proventricular glands (Pg). (H& E). (**C**) Photomicrograph of a section in the proventriculus of a 15 day old quail embryo shows the tip of the mucosal fold (Mf) is lined with simple columnar epithelium, which becomes simple cuboidal (arrows) towards the base of the fold. Lumen (Lum), connective tissue core of the fold (*), and lamina propria (Lp). (H& E). (**D**) Photomicrograph of a section in the proventriculus of a 15 day old quail embryo showing complete canalization of the simple tubular glands (arrows). Lumen (Lum), lamina properia (Lp), inner layer of muscularis mucosae (arrowhead), and proventricular glands (Pg). (H& E). (**E**) Photomicrograph of a section in the proventriculus of a 15-day-old quail embryo showing the simple tubular glands (Sg) are lined with simple cuboidal epithelium and open into the base of the sulci (*) between the mucosal folds (Mf). Notice that the simple tubular glands are located in the lamina propria (Lp), superficial to the discontinuous inner layer of the muscularis mucosae (arrow). Lumen (Lum). (H& E). (**F**) Photomicrographs of sections in the proventriculus of a 15-day-old quail embryo show the secretory units (Su) of the compound glands are lined solely with simple cuboidal epithelium. connective tissue septa (*) (H& E).

The tip of the mucosal fold was lined with simple columnar epithelium, which transformed into simple cuboidal toward the base of the fold. In some areas, additional folds and sulci were arranged concentrically around the glandular duct opening, giving the mucosal papilla a spiral appearance (Fig. 17A–C).Many of the simple tubular glands showed complete canalization, lined with simple cuboidal epithelium and opened into the base of the sulci between the mucosal folds. They were located in the lamina propria, superficial to the discontinuous inner layer of the muscularis mucosae (Fig. 17D,E).

The secretory units of the compound glands became lined solely with simple cuboidal epithelium (oxyntico-peptic cells), and the apical portion of the cells appeared regular. The lumina of the secretory units were free from cellular debris (Fig. 17F).

### Seventeen-day embryo

At this age, the four tunics constituting the wall of the proventriculus became well established as tunica mucosa, very thin tunica submucosa, tunica muscularis, and outer most tunica serosa. The two types of proventricular glands situated within the tunica mucosa.

**Figure 18:**
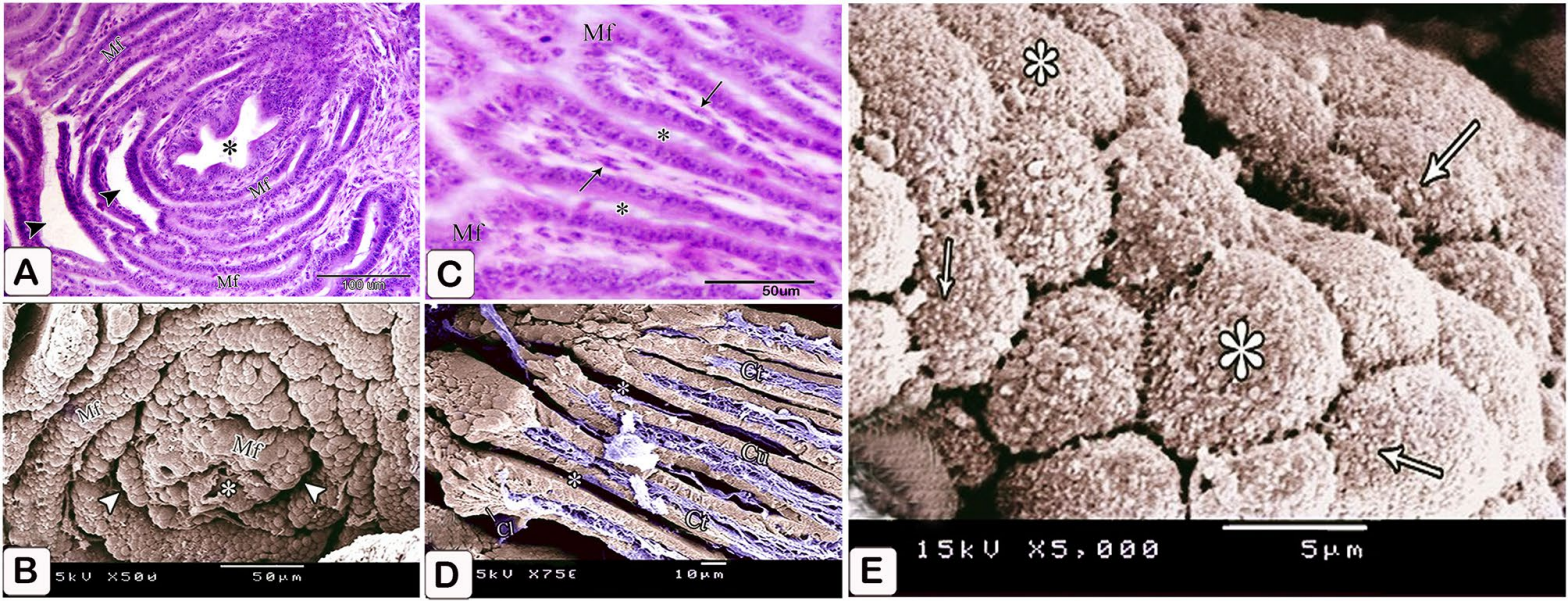
Photomicrographs of paraffin sections and digital coloring of a scanning electron micrograph of a 17-day-old quail embryo. (**A**) Photomicrograph of a section in the proventriculus of a 17-day-old quail embryo showing the addition of more folds to the mucosal papilla, giving it a definite whorled appearance. Mucosal folds (Mf), intervening sulci (arrowheads), and opening of the proventricular gland (*). (H & E). (**B**) digital coloring of a scanning electron micrograph of the luminal surface of the proventriculus of a 17-day-old quail embryo showing the whorled appearance of the mucosal papilla. Mucosal folds (Mf), intervening sulci (arrowheads), and opening of the proventricular gland (*) (**C**) Photomicrograph of a section in the proventriculus of a 17-day-old quail embryo showing the much higher mucosal folds (Mf) and the deeper sulci (*). Notice the numerous smooth muscle fibers (arrows) within the connective tissue core. (H & E). (**D**) Digital coloring of a scanning electron micrograph of the proventriculus of a 17-day-old quail embryo showing simple columnar epithelium (Cl) lining the top of the mucosal fold while the base of the fold is lined with simple cuboidal epithelium (Cu). Connective tissue core (Ct) and intervening sulci (*). (**E**) digital coloring of a scanning electron micrograph of the luminal surface of the proventriculus of a 17-day-old quail embryo showing the lining epithelial cells (*) are polygonal in shape with numerous short microvillus-like structures (arrows).

The light and scanning electron microscopic results revealed that more folds were added to the concentric plical structure of the mucosal papilla, giving it a definite whorled appearance (Fig. 18A,B). The plicae became much higher than that of the previous age and the sulci became much deeper. The tip of the fold was lined with simple columnar epithelium, which decreased in height to be transformed into simple cuboidal toward the base of the fold. The folds’ connective tissue core was densely packed with smooth muscle fibers and telocytes (Fig. 18C,D). The lining epithelial cells' luminal surface was polygonal in form, with numerous short microvillus-like features. (Fig. 18E).

**Figure 19:**
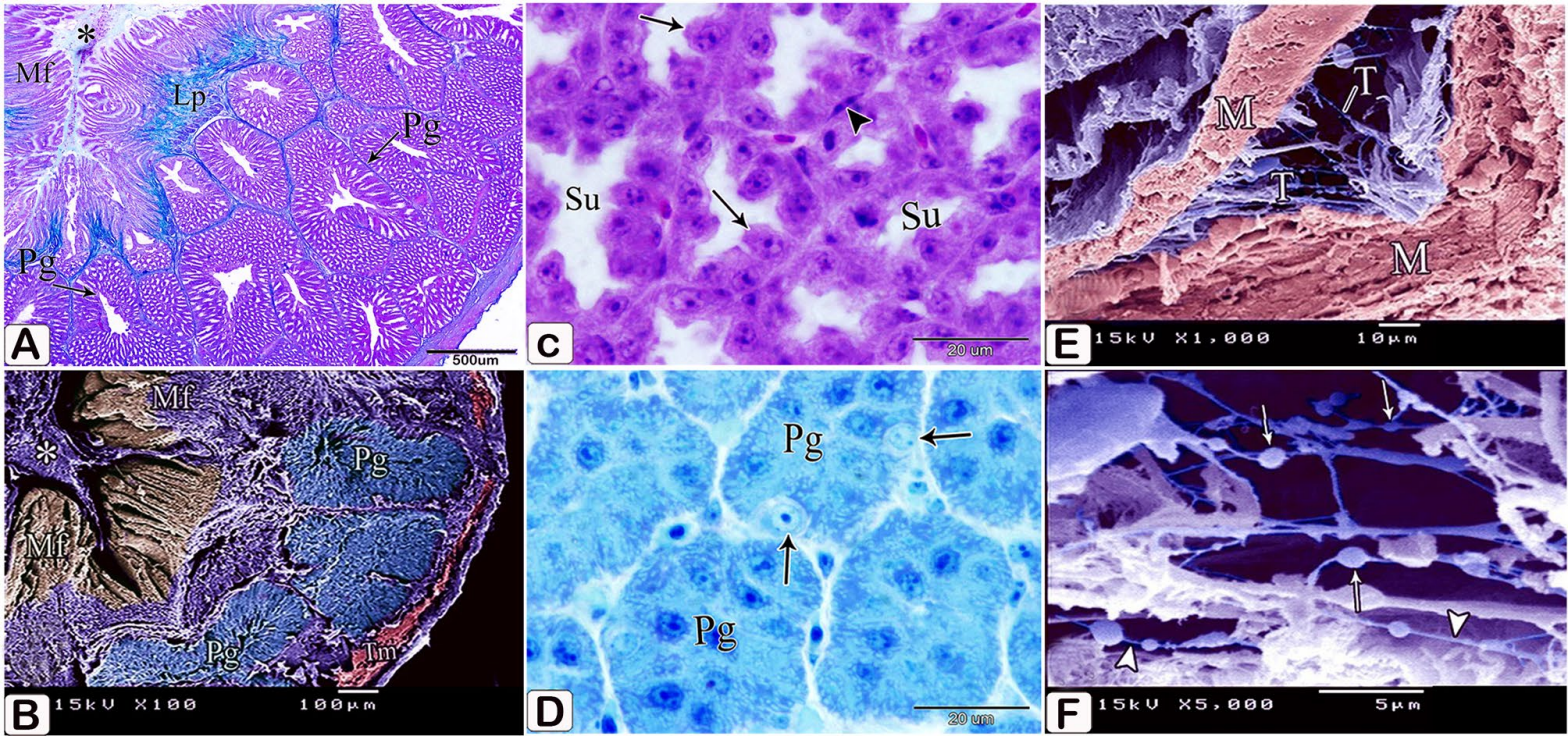
Photomicrographs of paraffin and semithin sections and digital coloring of a scanning electron micrograph of a 17-day-old quail embryo. (**A**) Photomicrograph of a section in the proventriculus of a 17-day-old quail embryo showing the compound proventricular glands (Pg) are much more branched, forming most of the thickness of the wall. Notice that the gland lobules are tightly packed together and separated only by a very thin layer of connective tissue (arrows). Lumen (*), mucosal folds (Mf), and lamina propria (Lp) (Crossmon’s trichrome stain). (**B**) Scanning electron micrograph of the proventriculus of a 17- day-old quail embryo showing the compound proventricular glands (Pg) that form most of the thickness of the wall. Lumen containing mucus (*), mucosal folds (Mf), and tunica muscularis (Tm). (**C**) Photomicrograph of a section in the proventriculus of a 17-day-old quail embryo showing dentate appearance (arrows) of the lining epithelium of the secretory units (Su) of the compound proventricular glands Notice that smooth muscle fibers (arrowheads) surround the secretory units. (H&E). (**D**) Photomicrograph of a semithin section in the proventriculus of a 17-day-old quail embryo showing closed-type endocrine cells (arrows) in between the glandular epithelium of the proventricular glands (Pg). (Toluidine blue). (**E**,**F**) Scanning electron micrographs of the proventriculus of a 17-day-old quail embryo showing a network of telocytes (T) between the muscle fibers (M). Notice the beaded appearance of the telopodes. Podoms (arrows) and podomeres (arrowheads).

Most of the wall’s thickness is now made up of the branched compound proventricular glands. The gland lobules were close to each other and were only divided by a very thin layer of smooth muscle fiber-filled connective tissue that wrapped around the secretory units. (Fig. 19A,B). Moreover, at this age, some of the epithelial cells lining the secretory units made contact with the adjacent cells only at their basal portions, giving the lining epithelium a dentate appearance while the apical portion of other cells remained regular (Fig. 19C). The glandular epithelium contained closed-type endocrine cells. (Fig. 19D).

Telocytes with long telopodes showing a beaded appearance were observed between the muscle fibers (Fig. 19E,F).

### Histochemical investigations

#### Neutral and acid mucopolysaccharides

**Figure 20:**
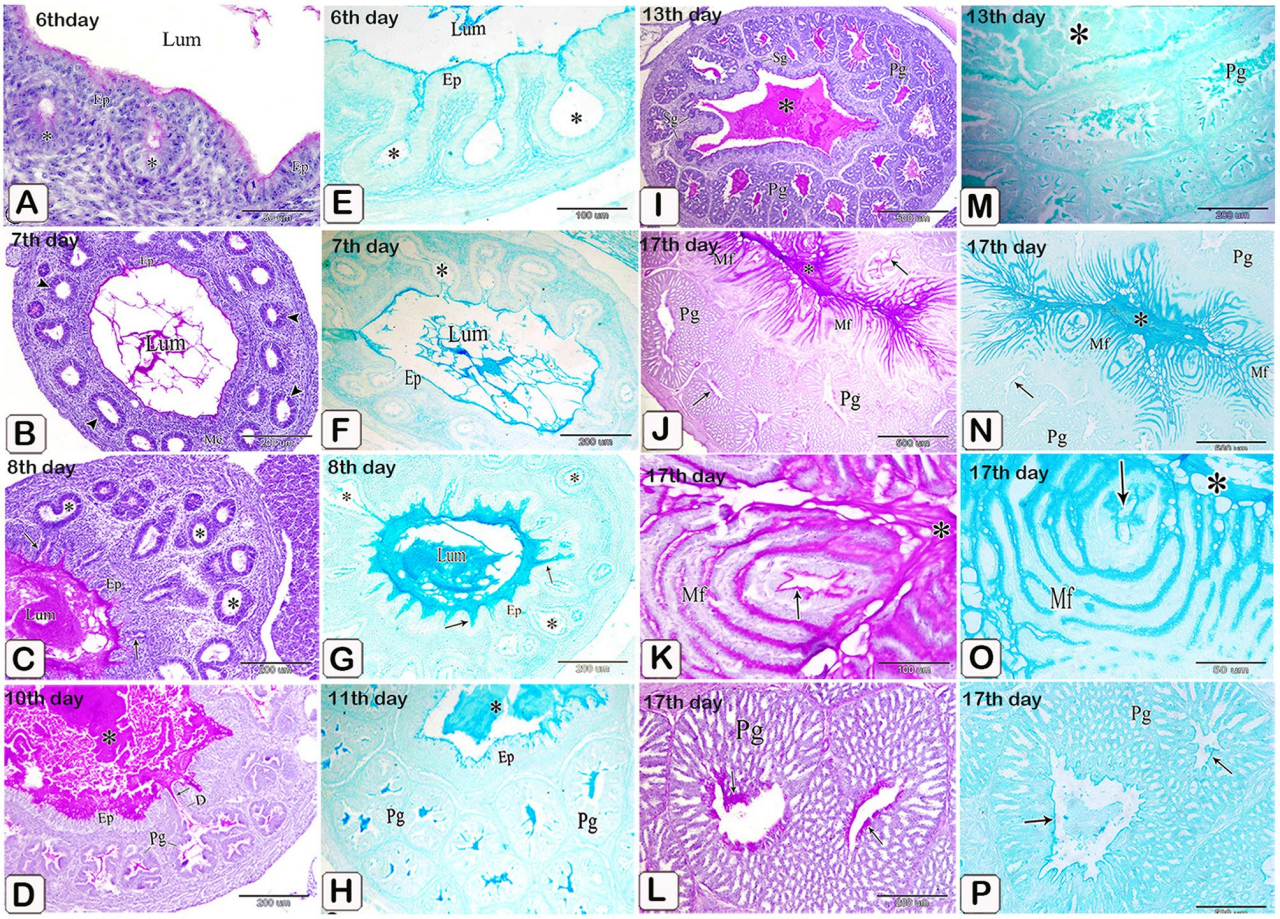
Photomicrographs of paraffin sections stained with PAS (**A**–**D**,**I**–**L**) and Alcian blue pH 2.5 (**E**–**H**,**M**–**P**) to show the presence of neutral and acid mucopolysaccharides in the proventriculus of quail embryos of various ages. (**A**,**E**,**B**,**F**) Photomicrographs of sections in the proventriculus of 6 (**A** and **E**) and 7 (**B** and **F**) day old quail embryos showing PAS and Alcian blue positive reactions in the epithelium (Ep) lining the lumen (Lum) of the proventriculus as well as the gland primordium (*). (**C**,**G**) Photomicrographs of sections in the proventriculus of an 8-day-old quail embryo showing strong positive reactions for PAS and Alcian blue in the luminal content (Lum), apical border of the luminal epithelium (Ep), and along the primary ducts of the glands (arrows). Notice, the gland primordium (*) shows weak reactions. (**D**,**H**,**I**,**M**) Photomicrographs of sections in the proventriculus of 10 (**D**), 11 (**H**), and 13 (**I**,**M**) day old quail embryos show strong positive reactions for PAS and Alcian blue within the luminal content (*), apical border of the luminal epithelium (Ep), and the simple tubular glands (Sg), while the epithelium of the compound proventricular glands (Pg) shows negative reactions. Notice that mucus strands (arrows) possessing positive PAS and Alcian blue reactions extend from the lumen of the proventriculus into the ducts of the glands (**D**). (**J**,**K**,**L**,**N**,**O**,**P**) Photomicrographs of paraffin sections in the proventriculus of a 17-day-old quail embryo showing the secretory products filling the apical portions of the epithelial cells lining the mucosal folds (Mf) and the intraluminal mucus coat (*) are strongly stained with PAS and Alcian blue. Notice, that the ductal epithelium (arrows) shows positive reactions while the secretory units of the compound proventricular glands (Pg) are negative for both reactions.

The epithelium lining the lumen of the proventriculus as well as the gland primordium showed strong PAS and moderate Alcian blue positive reactions on the 6th and 7th day of incubation (Fig. 20A,B,E,F). On the 8th day, strong positive reactions for PAS and Alcian blue were observed in the luminal content, apical border of the luminal epithelium, and along the primary ducts of the glands, whereas the gland primordium showed weak reactions (Fig. 20C,G). In older embryos, from 9 to 17 days (Fig. 20D,H,I–P), the glandular epithelium showed negative PAS and Alcian blue reactions. Moreover, mucus strands showing positive PAS and Alcian blue reactions extended from the lumen of the proventriculus into the ducts of the glands. The simple tubular glands gave strong positive reactions for PAS and Alcian blue from the 12 and 13th day of incubation (Fig. 20I,M). On the 17th day, the secretory products filling the apical portions of the epithelial cells lining the mucosal folds as well as the intraluminal mucus coat were strongly stained with PAS and Alcian blue. These products decreased in amount toward the base of the folds. The ductal epithelium showed positive PAS and Alcian blue reactions, whereas the epithelium lining the secretory units of the compound proventricular glands was negative for both reactions (Fig. 20J,K,L,N,O,P).

#### Protein

**Figure 21:**
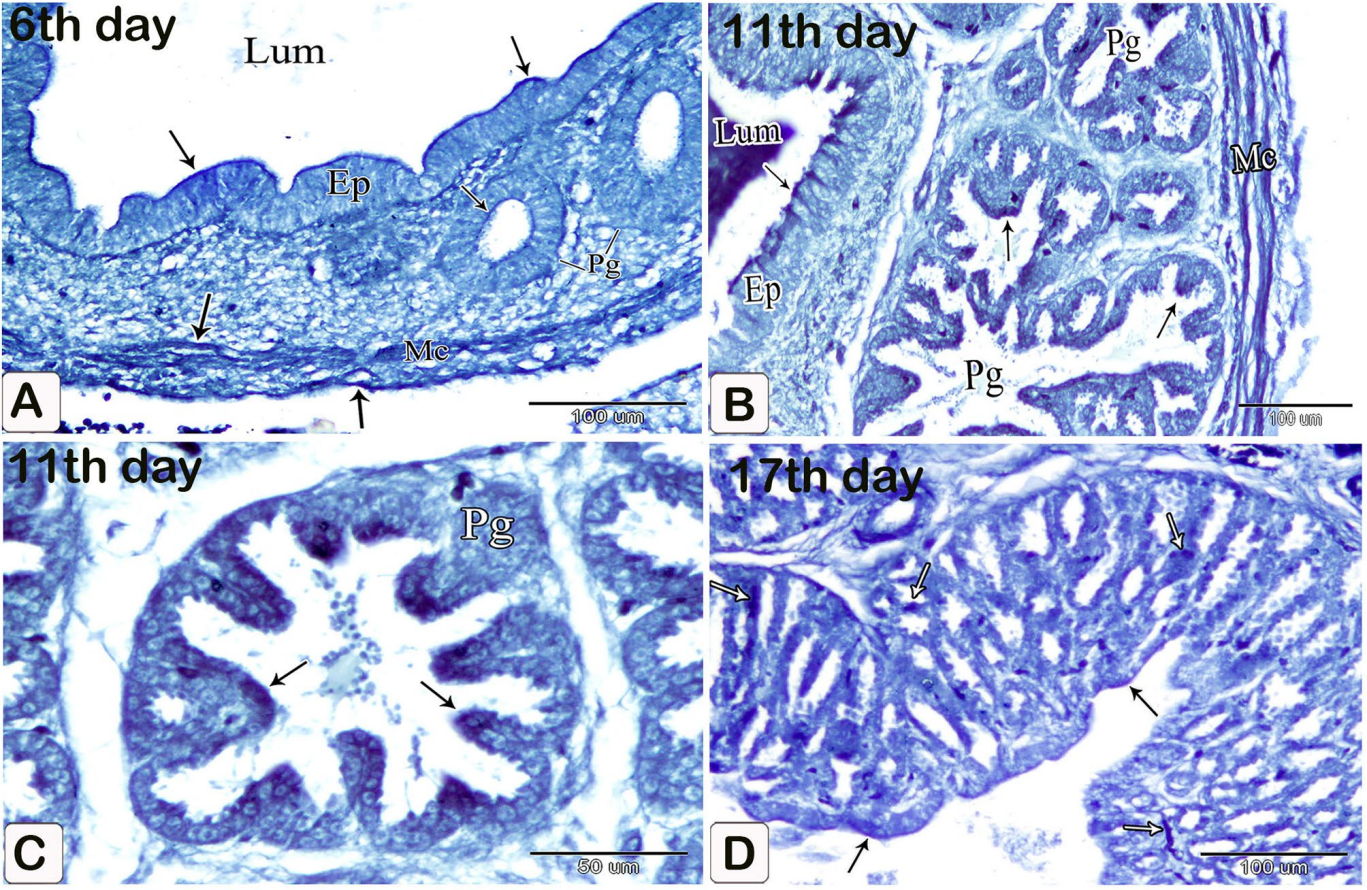
Photomicrographs of paraffin sections in the proventriculus of 6 (**A**), 11 (**B** and **C**), and 17 (**D**) day old quail embryos showing positive reactions (arrows) for bromphenol blue in the apical portion of the luminal and glandular epithelium as well as the muscular coat and the mesothelium. Lumen (Lum), luminal epithelium (Ep), proventricular glands (Pg), and muscular coat (Mc) (Mercury bromphenol blue stain).

The apical portion of the luminal and glandular epithelium as well as the muscular coat and the mesothelium was stained with mercury bromphenol blue (intense blue) which increased in strength from day 6 to day 17 of incubation (Fig. 21A–D).

#### Acridine orange

**Figure 22:**
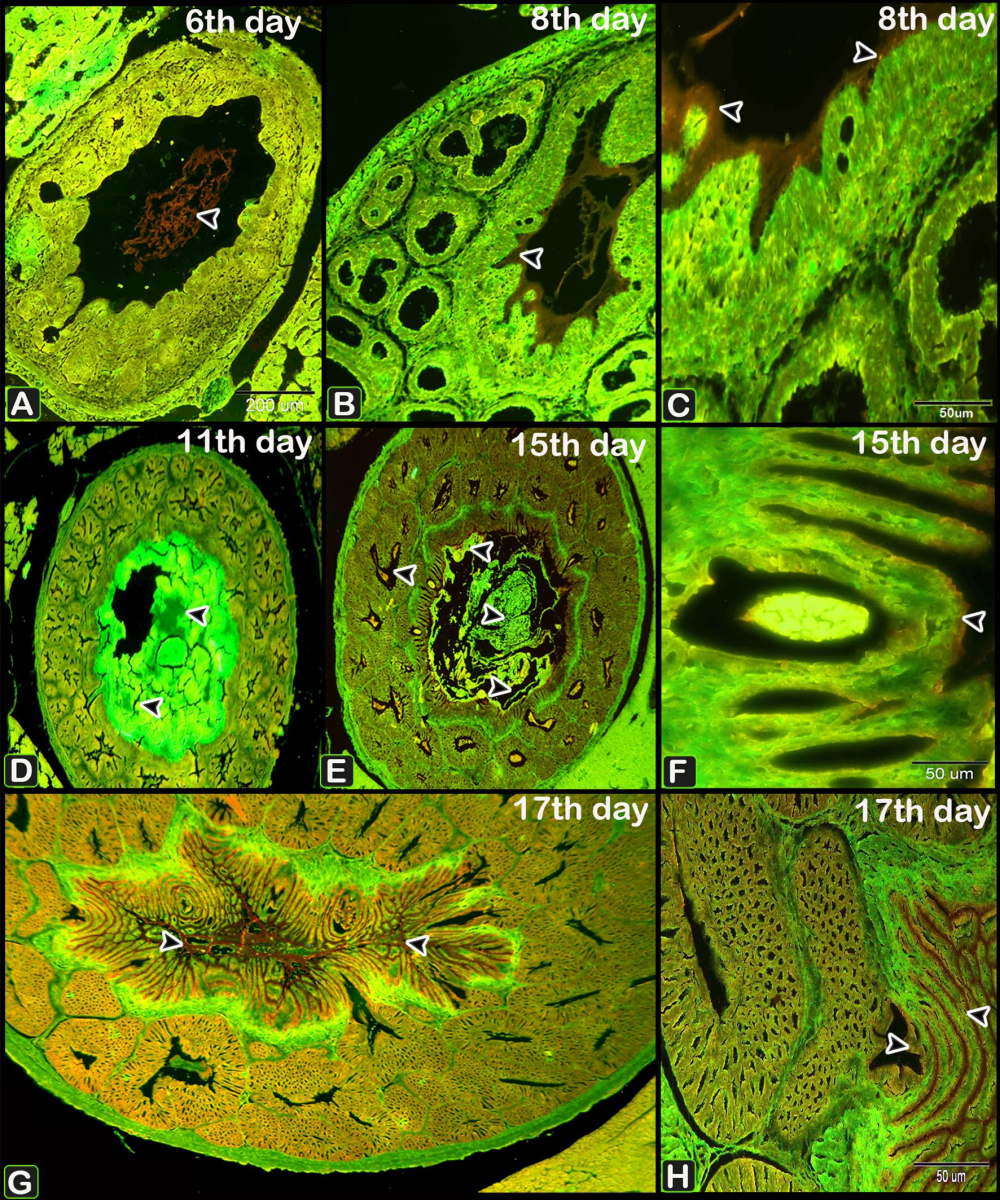
Photomicrographs of paraffin sections stained with acridine orange in the proventriculus of 6 (**A**), 8 (**B**,**C**) and 11 (**D**), 15 (**E**,**F**), and 17 (**G**,**H**) day old quail embryos showing increased acidic reactions (arrowheads) in the apical area of the luminal and glandular epithelium, as well as secretory products filling the apical portions of epithelial cells lining the mucosal folds.

From day 6 to day 17 of incubation., acidic secretions in the apical area of the luminal and glandular epithelium, as well as secretory products filling the apical portions of epithelial cells lining the mucosal folds increased (Fig. 22A–H).

#### Enzymatic activity

**Figure 23:**
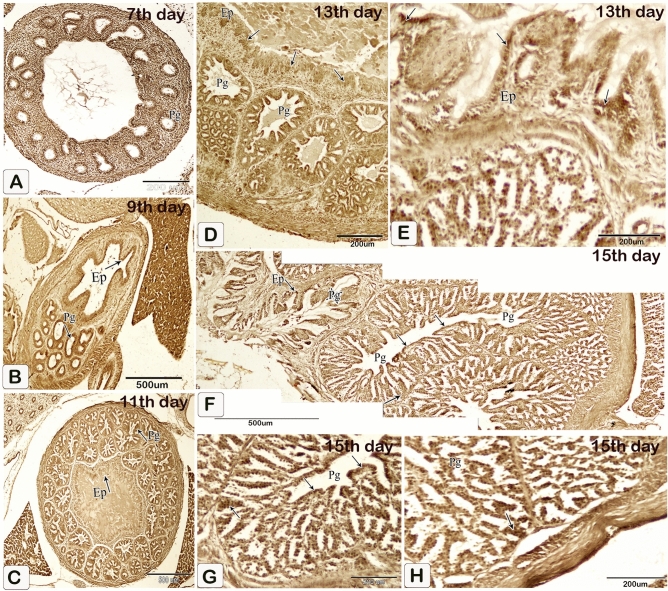
Photomicrographs of frozen sections in the proventriculus of 7 (**A**), 9 (**B**), 11 (**C**), 13 (**E** and **D**), and 15 (**F**–**H**) day old quail embryos showing positive reactions (arrows) for alkaline phosphatase enzymes within the luminal and glandular epithelium as well as the epithelium lining the ducts. luminal epithelium (Ep) and proventricular glands (Pg).

**Figure 24:**
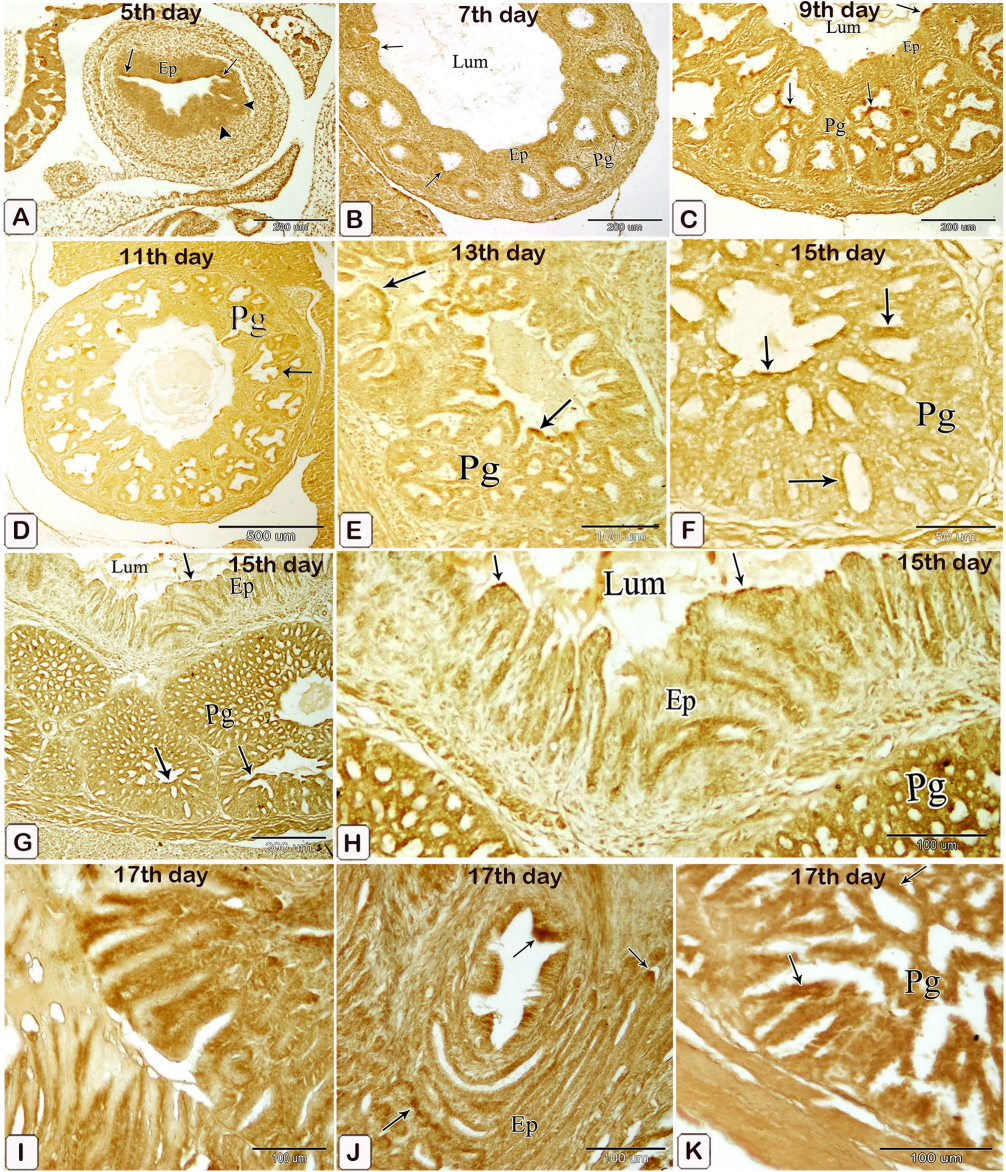
Photomicrographs of frozen sections in the proventriculus of 5 (**A**), 7 (**B**), 9 (**C**), 11 (**D**), 15 (**E**), 15 (**F**–**H**), and 17 (**I**–**K**) day old quail embryos showing positive reactions (arrows) for acid phosphatase enzymes within the luminal and glandular epithelium as well as the epithelium lining the ducts. Luminal epithelium (Ep) and proventricular glands (Pg).

**Figure 25:**
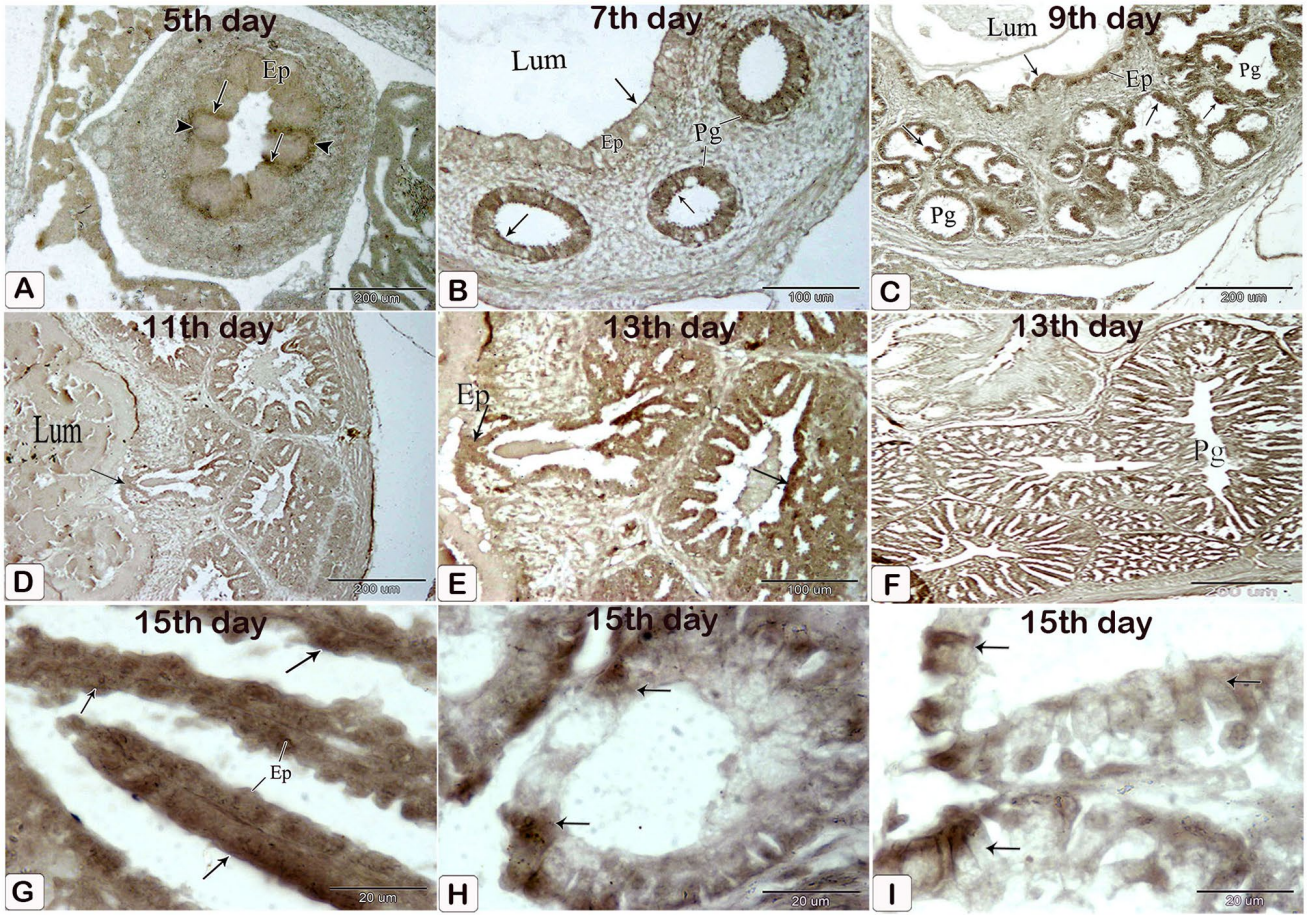
Photomicrographs of frozen sections in the proventriculus of a 5-day-old quail embryo showing weak positive reactions (arrows) for ATPase enzymes within the luminal epithelium (Ep) and the epithelium lining the gland rudiments (arrowheads). (**A**)5,  (**B**)7, (**C**) 9, (**D**) 11, (**E**,**F**) 13, and (**G**–**I**)15 day old quail embryos showing positive reactions (arrows) for acid phosphatase and ATPase enzymes within the luminal and glandular epithelium as well as the epithelium lining the ducts. Lumen (Lum), luminal epithelium (Ep), and proventricular glands (Pg).

The luminal epithelium and epithelium lining the gland rudiments gave weak positive reactions for alkaline and acid phosphatase and ATPase enzymes on the 5th day of incubation. These reactions increased as fetal age increased from the seventh day to the end of incubation including the luminal and glandular epithelium as well as the epithelium lining the ducts (Figs. 23, 24, 25).

## Macroscopic investigations

**Figure 26:**
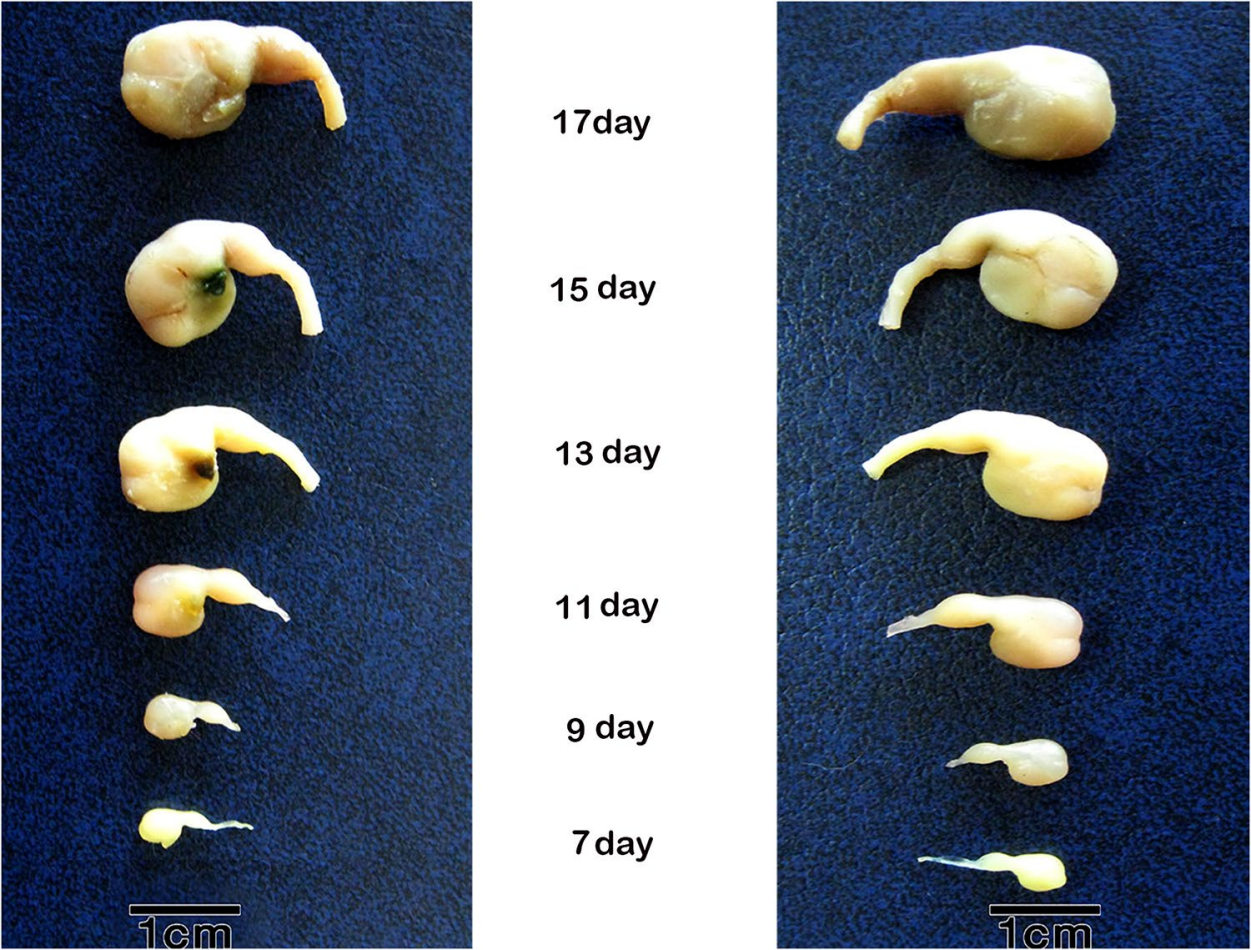
Photograph of the proventriculus (P) of quail embryos at different stages of development, showing the spindle shaped proventriculus between the esophagus (E) and the gizzard (G).

The form of the glandular stomach did not alter much from the 7th day to the end of incubation. It had a spindle-shaped appearance, with a larger caudal end separated from the muscular stomach by an isthmus, and a little constriction separating it from the esophagus (Fig. 26).

The weight of the glandular stomach was about 0.005 gm at 7 days old embryo, and it continuously increased until the end of the incubation period, when it reached about 0.052 gm at 17 days old embryo. The glandular stomach formed around 21% of the weight of the stomach complex at 7 days old embryo, and this percentage decreased until it reached its lowest point at 17 days old embryo, when it was about 13.5% of the weight of the stomach complex (Table 1).

**Table 1:** Weight of the stomach complex, glandular stomach and the ratio between them.

Age of embryo (days)	Weight of stomach complex (mg) (W.S.C)	Weight of glandular stomach (mg) (W.G.S)	Ratio of (W.G.S) to (W.S.C) (%)
7	0.024 ± 0.001	0.005 ± 0.002	20.83
11	0.11 ± 0.017	0.022 ± 0.003	20.00
13	0.19 ± 0.055	0.024 ± 0.007	12.63
15	0.30 ± 0.065	0.045 ± 0.011	15.00
17	0.38 ± 0.035	0.052 ± 0.002	13.68

All weight values are multiplied by 10^3^.

Values are represented by means ± standard deviation (SD).

The length and width of the glandular stomach measured about 1.74 and 1.35 mm at 7 day old embryo respectively. Then the two parameters increased with the advancement of age until reached its maximum value on the 17th day being about 4.25 and 3.89 mm, respectively (Table 2).

**Table 2:** Length and width of the glandular stomach at different stages of development.

Age of embryo (days)	Mean ± SD	
	Glandular stomach (mm)	
	Length	Width
7	1.74 ± 0.22	1.35 ± 0.07
11	3.55 ± 0.39	2.92 ± 0.09
13	3.72 ± 0.27	3.04 ± 0.12
15	3.79 ± 0.56	3.47 ± 0.28
17	4.25 ± 0.29	3.89 ± 0.66

## Conclusion of main age changes

**Figure 27:**
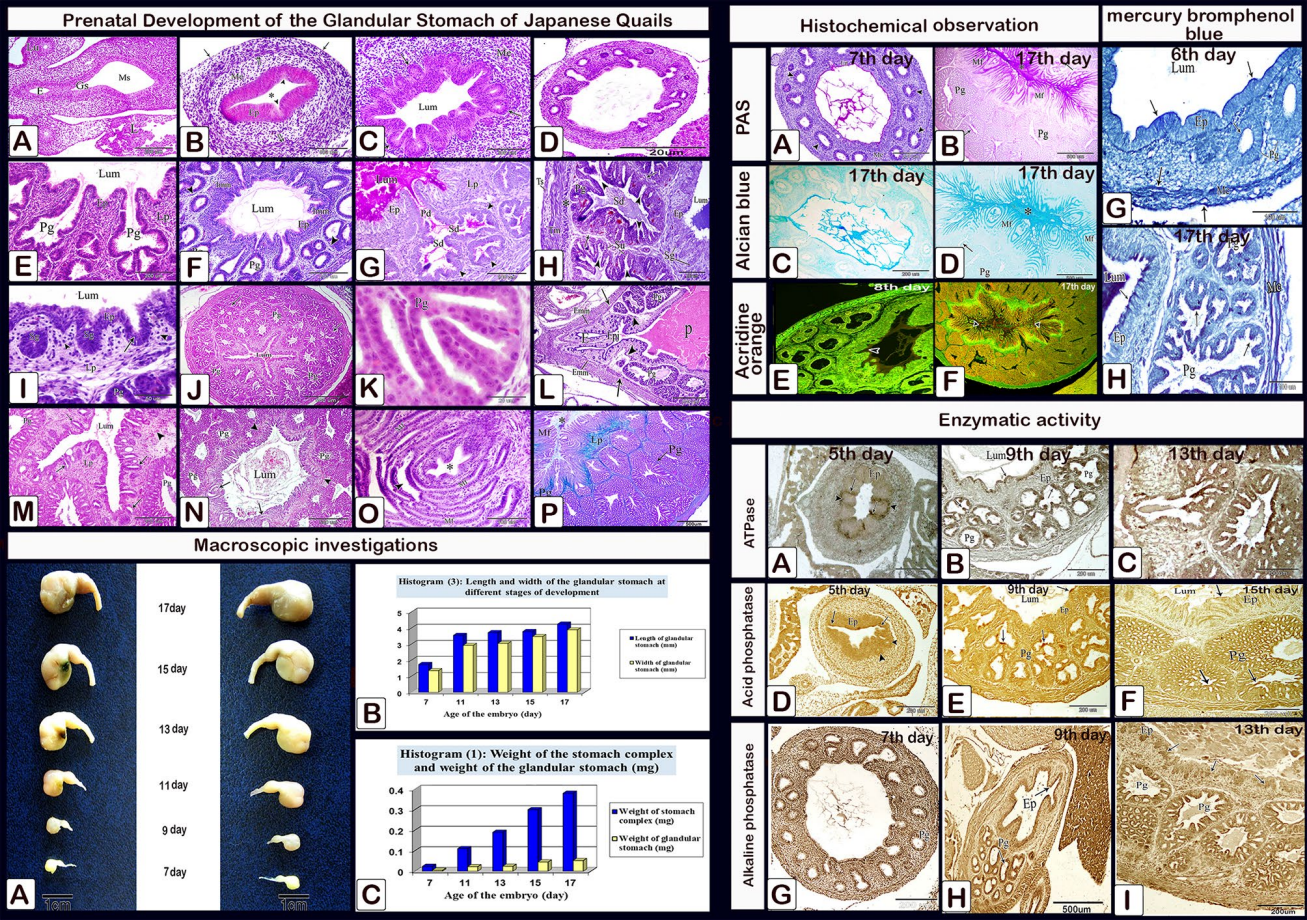
Under the microscope, the Prehatching development of the glandular stomach of Japanese Quails. (**A**) 3-day-old quail embryo showing the prospective glandular stomach (Gs) is continued with the esophagus (E) cranially and the prospective muscular stomach (Ms) caudally. Lung (Lu) and liver (L). (**B**) Sections in the proventriculus of a 4-day-old quail embryo showing the primordium of the proventricular glands (arrowheads). Epithelium (Ep), mesenchyme (Me), mesothelium (arrows), and lumen (*). Notice the condensation of the mesenchyme (double arrows) nearly in the middle of the mesenchymal layer. (**C**) The proventriculus of a 5-day-old quail embryo showing the gland primordium (arrows) spreading throughout most of the mucosa. Mesenchyme (Me) and lumen (Lum) (**D**) proventriculus of a 6-day-old quail embryo showing varying degrees of development in the proventricular glands (*). Lumen (Lum), luminal epithelium (Ep), mesenchyme (Me), muscular coat (Mc), and mesothelium (arrowhead). (**E**) An 8-day-old quail embryo showing the proventricular mucosal surface exhibits many small folds (Mf) with intervening depressions (arrows). Notice, condensation of the mesenchyme (*) under the luminal epithelium (Ep) and the branching of the proventricular glands (Pg). Lumen (Lum), outer layer of muscularis mucosae (arrowheads), tunica muscularis (Tm), and liver (L). (**F**) An 8-day-old quail embryo showing differentiation of the muscular coat. Lumen (Lum), luminal epithelium (Ep), proventricular glands (Pg), lamina propria (Lp), inner muscularis mucosae (Imm), outer muscularis mucosae (Omm), inner tunica muscularis (Itm), outer tunica muscularis (Otm), tunica submucosa (*), and tunica serosa (Ts). Notice, few muscle fibers (arrowheads) extend between the glands. (**G**) The proventriculus of a 10-day-old quail embryo shows division of the primary duct (Pd) of the proventricular gland into two secondary ducts (Sd). Secretory units (arrowheads), lumen (Lum), luminal epithelium (Ep), and lamina propria (Lp) (PAS). (**H**) The proventriculus of an 11 day old quail embryo shows the proventricular glands (Pg) as compound tubuloalveolar glands where the secretory units (Su) are connected to the secondary ducts (Sd) by tertiary ducts (arrowheads). Lumen (Lum), luminal epithelium (Ep), primordium of the simple tubular glands (Sg), connective tissue septa (arrows), outer muscularis mucosae (*), tunica muscularis (Tm), and tunica serosa (Ts). (**I**) The proventriculus of an 11-day-old quail embryo shows the primordium of the simple tubular glands (Sg) as localized down-growths of the luminal epithelium (Ep) into the lamina propria (Lp) forming solid cords. Lumen (Lum) and proventricular glands (Pg). (**J**) The proventriculus of a 12-day-old quail embryo shows the compound tubuloalveolar glands (Pg) are more branched, and most of their lining epithelium is of low columnar type. Notice that the connective tissue septa (arrows) separating the gland lobules became thinner. Lumen (Lum). (**K**) the proventriculus of a 12-day-old quail embryo showing the lamina propria (Lp) and the compound glands (Pg). Notice the increased number of simple tubular glands (Sg) and that very few of them begin to be canalized (arrow). Lumen (Lum), secretory units (Su), secondary ducts (Sd), and tertiary ducts (Td) (**L**) 13-day-old quail embryo showing the muscularis mucosae of the esophagus (Emm) diverge at the esophago-proventricular junction (Epj) into inner and outer layers that continue with the inner (arrowheads) and outer (arrows) layers of the muscularis mucosae of the proventriculus (**P**), which re-join prior to the isthmus (*) to continue with the muscularis mucosae of the gizzard (double arrows). Proventricular glands (Pg), gizzard (G), and liver (L). (**M**) The proventriculus of a 15 day old quail embryo shows complete canalization of the simple tubular glands (arrows). Lumen (Lum), lamina properia (Lp), inner layer of muscularis mucosae (arrowhead), and proventricular glands (Pg). (**M**) The proventriculus of a 15-day-old quail embryo showing complete canalization of the simple tubular glands (arrows). Lumen (Lum), lamina properia (Lp), inner layer of muscularis mucosae (arrowhead), and proventricular glands (Pg). (**N**) The proventriculus of a 15-day-old quail embryo showing the spiral appearance of some mucosal papillae (arrows). Lumen (Lum), inner layer of muscularis mucosae (arrowhead), and proventricular glands (Pg). (**O**) The proventriculus of a 17-day-old quail embryo shows the addition of more folds to the mucosal papilla, giving it a definite whorled appearance. Mucosal folds (Mf), intervening sulci (arrowheads), and opening of the proventricular gland (*). (**P**) The proventriculus of a 17-day-old quail embryo shows the compound proventricular glands (Pg) are much more branched, forming most of the thickness of the wall. Notice, the gland lobules are tightly packed together and separated only by a very thin layer of connective tissue (arrows). Lumen (*), mucosal folds (Mf), and lamina propria (Lp). Histochemical investigations: From the 6th to the 17th day of incubation, the intensity of reactions to alkaline, acid phosphatase, and ATPase enzymes, mercury bromphenol blue, PAS, and Alcian blue increased.

Figure 27 depicts the major morphological changes.

## Discussion

Development of the chick embryo’s stomach complex began on the third day of incubation with the establishment of an expansion of the foregut directly anterior to the hepatic diverticula^44^. However, in the present work, the primordium of the stomach in the quail could be identified on the 2nd day of incubation. Based on our findings and that of Attia^43^ the stomach could be distinguished into two parts: a small cranial part **(**prospective glandular stomach**)** and a large caudal part **(**prospective muscular stomach**)** on the 3rd day of incubation. In the chick embryo, the proventriculus could be identified from the gizzard on the 6th day^45^. However, on the tenth day of incubation, the two sections of the stomach in the helmeted guinea fowl could be distinguished from one another^46^. The development and differentiation of the epithelium in the chick proventriculus were dependent on inductive signals from the underlying mesenchyme^47^. In this respect, Yasugi^48^ and Roberts^49^ stated that, in birds, the epithelial-mesenchymal interaction was essential for normal gut development. Moreover, in the present study as well as that of Gallagher^50^, epithelial-mesenchymal contact was probably enhanced in areas where the basement membrane became thin or discontinuous. The lining epithelium of the primitive quail proventriculus was pseudostratified and changed into the simple columnar type by the 10th day of incubation in the current investigation. Attia^43^ as well as Soliman et al.^30^ observed in the same bird that the epithelial transformation occurred on Day 9 and 12, respectively. Different sources of fertilized eggs and incubation methods were postulated by later authors as a possible explanation for such contradictory findings. The light and scanning electron microscopical results revealed that many small mucosal folds with intervening depressions were observed for the first time in an 8-day-old embryo. These folds increased in number and height with the advancement of age. This is consistent with the results obtained by Martínez et al.^51^ in a chick embryo at Day 14 as well as Wali et al.^52^ at Day 16. The projection of the mucosa into numerous folds leads to the increase in the surface area and consequently the amount of mucin secreted by their lining cells in order to give more protection against the harmful effects of the gastric juice and ingested materials^26^.

Numerous papillae could be observed on the proventriculus’s mucosal surface on the 11th day. These papillae were formed by the connection of two folds after approaching each other around the opening of the proventricular glands. Similar papillae could be detected in the chick embryo on the 14th day^51^. With the advancement of age, some mucosal folds were concentrically arranged around the glandular duct openings, giving the mucosal papilla a definite whorled appearance on the 17th day. Stinson and Calhounin^53^ and Dellman^54^, as well as Salem et al.^25^ described that, in the chick, the mucosal folds had a concentric arrangement. Salem & Abdel-Rahman^26^ suggested that the concentric manner of arrangement of the mucosal folds around the openings of the proventricular glands probably gives better support and protection to the gland opening than the finger-like shapes. This is because the finger-like shapes could be damaged by the different ways this organ is stretched the concentric manner of arrangement of the mucosal folds around the openings of the prIn this work, the endocrine cells in the quail proventriculus were first seen on the 7th day of incubation between the luminal and glandular epithelium. However, Romanoff^18^ and Walter^55^, stated that in chick embryo, these cells began to appear in the epithelium of the lumen at embryonic day 8. They also saw that the number of endocrine cells in the main lumen epithelium decreased over time. These cells’ functions were mostly taken over by cells that appeared in the epithelium of the proventriculus glands on the 12th and 16th days of incubation, respectively. Yamaguchi et al.^56^ could observe in the quail proventriculus the embryonic endocrine cells for the first time on the 11th day of incubation in the superficial epithelium and proventricular glands.

According to the present study as well as that of Martínez et al.^51^, in the chick embryo, the open type of the endocrine cells was detected on day 7–12 or day 9–13 of incubation, respectively, after which their number decreased with the increased number of the closed type. Yamaguchi et al.^56^ observed in the quail proventriculus that no “open type” cells could be observed in all developmental stages. Moreover, Aksoy & Cinar^57^ observed in the chick embryo that both the gastrin and serotonin-IR cells were of the closed type in all stages studied.

In the present study, the proventricular glands had two types: superficial and deep (as previously reported by Langlois^58^ and Zaher, et al.^28^. The primordium of the compound deep proventricular glands in quail embryos appeared as evaginations of the lining epithelium on the 4th day of incubation. Similar results were observed in chick embryos around the 7th day of incubation^59^. It was unclear whether the proventricular glands lie in the lamina propria or within the submucosa. In the present study as well as that of Alsanosy et al.^33^on domestic fowls, Bezuidenhout, & Van Aswegen^60^ on ostriches, Van Alten& Fennell^61^ and Romanoff^18^ on chicken embryos, and Liman et al.^62^ on 1-day-old hatched chicks, the deep tubuloalveolar glands were located in the lamina propria. On the other hand, the studies of on fowls considered the glands to be submucosal Bradley, and Grahame^63^, Toner^64^.

Many authors have reported the fact that some change occurred in the glandular epithelium of the presumptive secretory unit of the proventriculus. The glandular epithelium changed from pseudostratified columnar to cuboidal epithelium on the 15th day of incubation in the chick embryo^57,59,65^ and in the helmeted guinea fowl^46^. Similarly, Attia^43^ stated that in the quail, the glands were still lined by stratified columnar epithelium and some glands were still undifferentiated and appeared as masses of stratified cells. However, Soliman, et al.^30^ in the same bird observed that on the 12th day of development, the pseudostratified epithelium of the glands transformed into a simple columnar type at the location of oxyntico-peptic cells. However, none of these investigators stated what actually happens. In the present study, the glandular epithelium of the quail embryos showed gradual changes from pseudostratified columnar on day 6 to simple columnar on day 12 and then into the established type on day 15 which was the simple cuboidal epithelium. In accordance with Thomson^66^, both light and electron microscopical observations of the proventricular glands of the studied bird revealed that the reorganization of the glandular epithelium occurred as follows: Firstly, by bulging of the apical portion of the superficial cells of the pseudostratified columnar epithelium into the lumen of the glands beyond the level of the terminal bars and their sloughing off, followed by the sloughing of the remaining part of the superficial cells. This was evidenced by the presence of cellular debris containing nuclei in the gland lumina. Secondly, by remolding of the basal cells from simple columnar to low columnar and finally to simple cuboidal.

Concerning the simple tubular glands, the present investigation revealed that their primordium could be detected on the 11th day as localized down-growths of the luminal epithelial cells into the lamina propria forming solid cords. These glands began to canalize on the 13th day. This was in accordance with that described in the chick embryo by Sjögren et al.^67^ in the lower and upper parts of the glandular stomach on the 11th and 13th day of incubation, respectively, as well as Martínez et al.^51^ on the 14th day. However, the studies of Bezuidenhout & Aswegen^60^ on ostriches, Ogunkoya & Cook^68^ on three species of Australian passerines, Liman et al.^62^ on 1-day-old chicks, and Le Guen et al.^69^ on gray-backed shrike stated that these glands formed by the invagination of the surface epithelial cells into the lamina propria. In earlier studies on domestic chickens, these glands were considered as artifacts of preparation^64^.

Our findings indicate that in 10-day-old embryos, the muscularis mucosae of the esophagus diverged at the esophago-proventricular junction into the thin inner and thick outer layers, which continued with the corresponding ones of the proventriculus. Similar results were reported by Czarnecki^70^ at 14 days of age and became more prominent on the 16th to 19th day. We also could observe the inner and outer layers of the muscularis mucosae rejoining prior to the isthmus, so we proposed that such arrangement of the muscularis mucosae probably plays apart in the emptying of the glands.

The histochemical observations revealed that the lining epithelium of the proventriculus showed strong PAS and moderate AB positive reactions on the 5th and 6th day of incubation, indicating its ability to produce both acidic and neutral mucin from these embryonic ages. With the advancement of age, these reactions became stronger in the lining epithelium as well as the primary ducts of the compound glands and the simple tubular glands from the 9th day till the day of hatching. These results were similar to that described by Hinsch^71^ in chick embryos**.** Ventura et al.^65^ stated that only the surface epithelium was strongly positive mainly on themost advanced stages**.** However, Soliman et al.^30^ mentioned that in quail embryos, the ductal epithelium of the proventricular glands showed no PAS and AB reactions from Day 12 to Day 17 of incubation.

The primordia of the compound glands showed strong PAS and moderate AB positive reactions on the 5th and 6th day of incubation; these reactions became weak on the 8th day and negative afterwards. This indicated that in the early stages of development, the glands could produce both acidic and neutral polysaccharides, whereas they originated from the luminal epithelium, keeping its same character. In the advanced ages, the compound glands became differentiated and hence could not produce neither acidic nor neutral polysaccharides. We agree on the importance of previous studies of Udoumoh et al. and Demirbağ et al.^72,73^. As a result, this study found The production of neutral and acidic mucins in the stomach at embryonic day 14 may reflect early secretory roles of glands in the stomach of broiler chicken. They suggested that morphological evidence of rapid development of broiler stomach glands in late pre hatch periods, as well as maturation of the glands at hatch. These modifications could be required for early food ingestion, digestion, and a strong innate immune response quickly after hatching.

Acridine Orange is a cationic dye that stains proteins-containing membranous vesicles, including as secretory vesicles, membrane-bound acidic compartments, and acidic lysosomes. The metachromatic reaction of Acridine Orange is connected with the liberation of green and red fluorescence. Acridine Orange reacted with membrane-bound vesicles to produce an orange or red colour. Secretory vesicles and lysosomes are identified with Acridine Orange^74,75^. Acidic secretions in the apical area of the luminal and glandular epithelium, as well as secretory products filling the apical portions of epithelial cells lining the mucosal folds, increased and were paraellel with an increase secretions of acidic mucin stained by alcian blue from the 6th to the 17th day of incubation.

The epithelium lining the ducts, as well as the proventricular mucosal epithelium and glands, showed positive ATPase, alkaline phosphatase, and acid phosphatase activity, similar to the findings of many previous studies^76–78^. We hypothesized that elevated enzymes, which were linked to an increase in development ages, might be required for early meal ingestion, digesting, and absorption.

In conclusion, in a two-day embryo, the stomach primordium was seen to be the most dilated region of the gut, dorsal and cranial to the liver primordium and ventral to the level of the notochord. An inner, thin endodermal epithelial lining and an outer, thick mesenchymal layer comprised the stomach wall. The quail proventriculus wall consists of four tunics on the seventh day of embryogenesis: tunica mucosa, very thin tunica submucosa, tunica muscularis, and the outermost tunica serosa. On the seventh day embryo the wall of the quail proventriculus consists of four tunics; tunica mucosa, very thin tunica submucosa, tunica muscularis and outer most tunica serosa. There are two types of proventricular glands; compound tubuloalveolar glands and simple tubular glands whereas, both types are situated within the tunica mucosa. The compound tubuloalveolar glands develop on the 4th day of incubation. Beginning as an evagination in the pseudostratified columnar epithelium lining the lumen of the proventriculus, the gland rudiment, by repeated bifurcation of the evaginated portion, by sloughing of the superficial cells, and by remodeling of the residual basal cells from columnar to cuboidal, becomes a multilobular compound tubuloalveolar gland composed of numerous secretory units. The simple tubular glands develop on the 11th day as localized down-growths of the luminal epithelial cells into the lamina propria forming solid cords which later become canalized. Figure 27 displays the primary morphological changes and serves as a conclusion to the preceding section.

## Materials and methods

### Ethical approval

The Committee for the Use and Care of Animal Experiments and the Faculty of Veterinary Medicine in Assiut, Egypt, also gave their approval to the project. The number of the ethics license (No. 06/2023/0046). All methods were done according to the relevant guidelines and regulations.

### ARRIVE guidelines

The research conformed to the Animals in Research: Reporting In Vivo Experiments (ARRIVE) guidelines^79^.

The nomenclature used in the present study was adapted to the Nomina Anatomica Avium^80^ as well as the available literature.

### Sample collection and fixation

In this study, 130 healthy Japanese quail (*Coturnix japonica*) eggs were used. The fertile eggs came from the Poultry Production Department at Assiut University’s Faculty of Agriculture. The incubation took place in an automatic incubator where the temperature (37˚C) and humidity (60–65%) were managed. From the second day of development until the eggs hatched (Table 3). Scarification occurred on days 15–17, when the skin was incised from the recti of the beak to the thoracic inlet, enabling access to the body cavity. The samples were gathered in conformity with Egyptian animal legislation and the Institutional Ethical Committee of Veterinary Medicine at Assiut University.

**Table 3:** Age and number of the used embryos.

Age (day)	Number of used embryos			
	Gross morphology	Light microscopy	Transmission electron microscopy	Scanning electron microscopy
2	–	9	–	–
3	–	10	–	–
4	–	9	–	–
5	–	6	–	–
6	–	6	–	2
7	3	5	2	–
8	–	8	–	2
9	1	7	–	–
10	–	5	–	–
11	3	5	2	2
12	–	6	–	–
13	3	6	2	–
15	3	6	3	–
17	3	6	3	2
Total	16	94	12	8

The eggshells were carefully cracked apart at the broad end, and the embryos that appeared to be healthy were carefully removed from their shells. The embryos were extracted from the eggs and processed as a whole for gross morphology and light microscopy. For electron microscopy, the embryos were dissected then the glandular stomach was obtained, opened, washed in normal physiological saline and small pieces were taken.

#### For gross morphological studies

**Figure 28:**
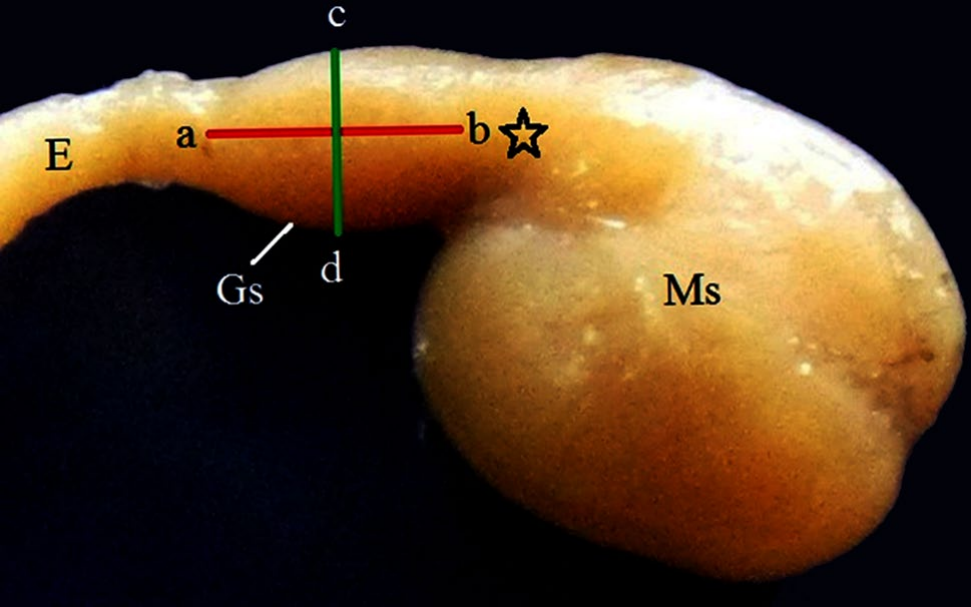
Method figure: Photograph of the stomach complex of a 17-day-old quail embryo showing the level of measurement of the length (ab) and width (cd) of the glandular stomach. Glandular stomach (Gs), esophagus (E), muscular stomach (Ms), and isthmus (*). MS (muscular stomach).

The quail embryos were fixed in 10% neutral buffered formalin then dissected for studying the length and width of the glandular stomach at different stages of development. The length of the glandular stomach (ab) was measured from the esophago-proventricular junction to the level of the beginning of the isthmus. The width (cd) was taken at the level of the midpoint of the length. After taking the images, these measurements were made using the Image J software (http://fiji.sc/Fiji, National Institutes of Health, USA).The weight of the stomach complex as well as the glandular stomach were recorded (The measurements are explained in the method Fig. 28). Stomach complex was described in Romanoff^18^.

#### For light macroscopically studies

Bouin’s fluid was used to preserve the embryos. After being dehydrated in a series of ethanol alcohol concentrations, the fixed specimens were cleared and embedded in paraffin wax (Table 4 displays the processing time for paraffin embedding). Transverse, sagittal, and frontal serial slices were cut at thicknesses of 3–5 µm using a Leica RM2125 microtome (Leica Microsystems, Wetzlar, Germany).For general histological studies, we used Harris haematoxyline and eosin stain; to demonstrate collagenous and muscle fibers, we used Mallory's trichrome stain; to identify such fibers, we used Crossmon’s trichrome stain; to detect acidic mucopolysaccharides, we used Alcian blue pH 2.5; and for neutral mucopolysaccharides, we used the PAS technique**.** Survan et al. described the staining procedures^81^. Mercury bromphenol blue stain for demonstration of protein^82^.

**Table 4:** The processing time of the samples in paraffin embedding techniques.

Age process	2d 3d 4d	5d 6d	7d 8d	9d 10d	11d 12d	13d	15d	17d
1-fixation	6 h	8 h	13 h	15 h	17 h	22 h	22 h	22 h
2-dehydration								
Alcohol 70% I	2 h	2 h	2 h	2 h	2 h	2 h	2 h	2 h
Alcohol 70% II	6 h	2 d	2d	2d	2d	7 h	1d	1d
Alcohol 70% III								
Alcohol 80%	1 h	1 h	2 h	2 h	2 h	2 h	2 h	2 h
Alcohol 90%	1 h	1 h	2 h	2 h	2 h	2 h	3 h	3 h
Alcohol 100%	10 min	1/2 h	1/2 h	1/2 h	3/4 h	1 h	1 h	1 h
Alcohol 100%	10 min	1/2 h	1/2 h	1/2 h	3/4 h	1 h	1 h	1 h
3-clearing with methylebenzot								
MBI	1 h	1 h	1 h	1 h	1 h	1 h	1 h	1 h
MB II	1d	1d	1d	1d	1d	1d	1d	1d
4-embedding in paraffin								
P I	1 h	2 h	2 h	2 h	2 h	2 h	2 h	2 h
P II	1 h	2 h	2 h	2 h	2 h	2 h	2 h	2 h
PIII	2,1/2 h	4 h	4h	6h	6h	18h	22h	22h

*h* hours, *d* days, *MB* I methyl bonzoate1, *MB* II methyl benzoate II, *PI* paraffin I, *P* II paraffin II, *P* III paraffin III.

### Use acridine orange dye to colour paraffin-cutting sections

The method used is based on the work of Hoff et al.^83^ and has been modified by^84^.

#### For enzyme histochemistry

For frozen sections, we take half-cm3 samples of each age. Following formal calcium fixation, samples were immersed in OCT overnight at 4 °C in the refrigerator and then frozen at − 20 °C for cryosectioning^85^ as directed by Suvarna et al.^81^.

The tissues were processed using the methods outlined by Suvarna et al.^81^ to identify the following enzymes: Acid phosphatase**,** Adenosine triphosphatase (ATPase), alkaline phosphatase. Leitz Dialux 20 microscope and Canon digital camera (Candison power shot A 95) were used to evaluate and take pictures of the produced sections.

#### For electron microscopy

Karnovesky’s solution (10 ml paraformaldehyde 25%, 10 ml gluteraldehyde 50%, 50 ml 0.1 M phosphate buffer PH 7.2, and 30 ml bidistilled water84) was used to fix small portions of the glandular stomach for 4–6 h at 4 °C. After washing the samples in a 0.1 M phosphate buffer, they were fixed in 1% osmium tetroxide for 2 h. The samples were then washed in the same buffer.

#### For semi thin section and transmission electron microscopy (TEM)

The fixed specimens were dehydrated in an increasing graded alcohol series before being imbedded in an Epon-Araldite combination, as described by Abd-Elhafeez et al.^86^, and as stated by Suvarna et al.^81^. Karnovsky’s Fixative^87^ was used to fix small sections of the specimens (2.0–3.0 mm length) at 4C overnight. The details of the process are explained in the Supplementary Materials. Semi-thin sections of 1 µm thickness were cut and stained with toluidine blue so that the specimens could be evaluated by light microscopy. The (600-800A°) ultrathin sections were stained with uranyl acetate and lead citrate^88^, investigated with a JEOL-100 CX II transmission electron microscope, and then photographed.

#### For scanning electron microscopy

Critical-point drying with liquid Co_2_ was used to dry the fixed tissues (0.5 × 0.5 cm^2^), after which they were dehydrated in ethanol and amyl acetate. They were sputter-coated with gold, placed on specimen stubs, and examined with a JEOL 5400 LV scanning electron microscope operating at 15 kV.

### Digitally colored scanning and transmission

Photoshop (Adobe, CS6) was used to digitally colour the scanning and TEM images, as described by^89^.

### Supplementary Information

Supplementary Information.

## Data Availability

On reasonable request, the corresponding author will provide the datasets used and/or analyzed during the current work.
