# Supplementary material for: Morphological studies on the prehatching development of the glandular stomach of Japanese quails using light, electron, and fluorescent microscopy
Source: Sci Rep. 2023 Oct 23;13:18096. doi: 10.1038/s41598-023-45355-1 (PMC10593961; doi:10.1038/s41598-023-45355-1)
Supplement: Supplementary file 1 — Supplementary Information. [file 41598_2023_45355_MOESM1_ESM.doc]

**Morphological Studies on the Prenatal Development of the Glandular Stomach of Japanese Quails Using Light, Electron, and Fluorescent Microscop**y

**Wafaa Gaber1, , Heba M. Mostafa1, Yousria A. Abdel-Rahman1, Hanan H. Abd-Elhafeez*2**

**1Department of Anatomy and Embryology, Faculty of Veterinary Medicine, Assiut University, Assiut, Egypt**

**2Department of cell and tissues, Faculty of Veterinary Medicine, Assiut University, Assiut, Egypt**

**Crosseponding author: Hanan H. Abd-Elhafeez***

[**hhnnzz91@aun.edu.eg**](mailto:hhnnzz91@aun.edu.eg)


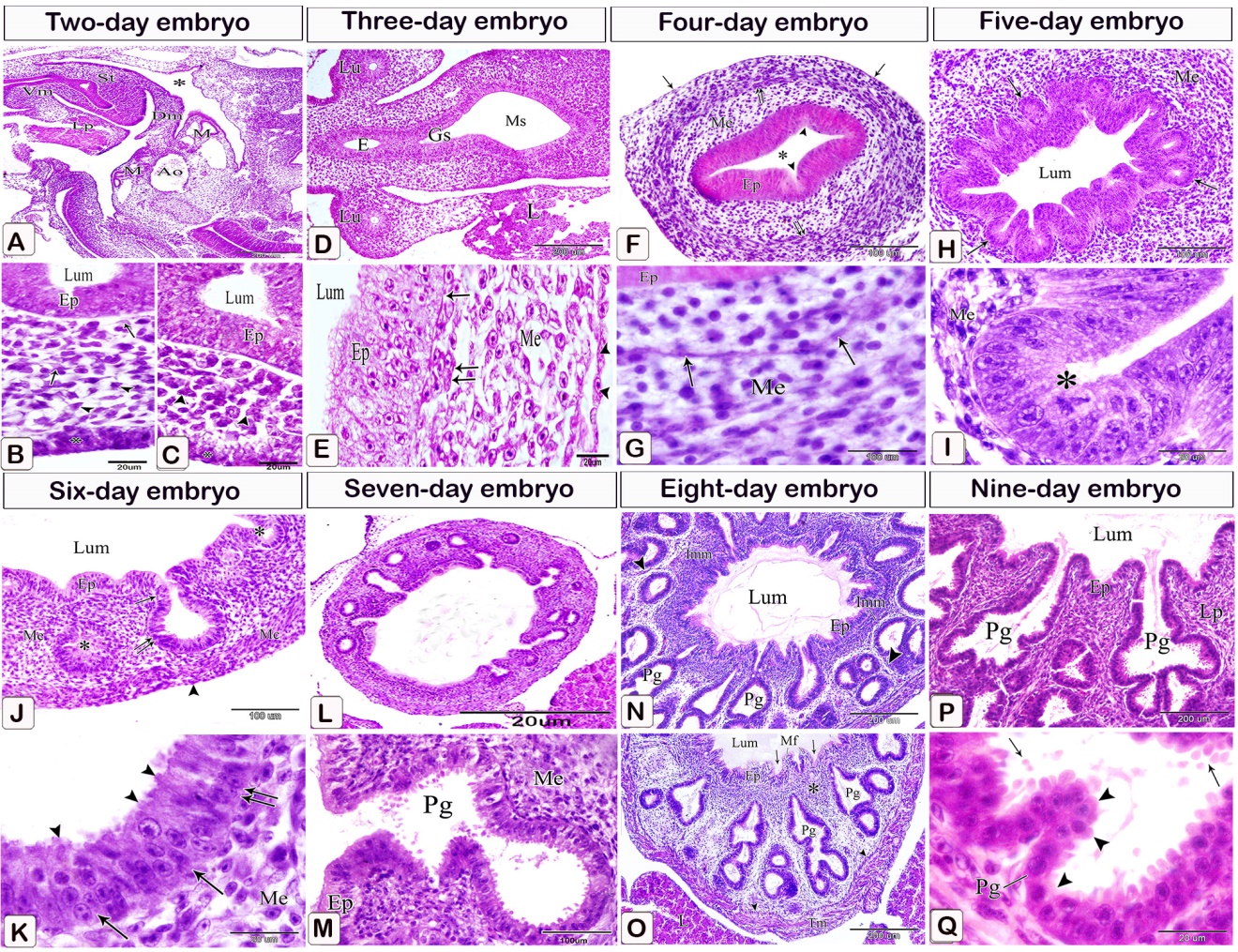


**Figure 1:**

**Two-day embryo**

The primordium of the stomach could be observed. The stomach wall was made up of an inner thin endodermal epithelial lining and an exterior thick mesenchymal layer (Figs.1 A, B, C).

**Three-day embryo**

The stomach could be distinguished into two parts: a small cranial part (prospective glandular stomach) and a large caudal part (prospective muscular stomach). The pseudostratified columnar epithelium rested on a discontinuous basement membrane. Some mesenchymal cells aggregated in groups under the epithelium. The mesothelium of the tunica serosa consisted of a single layer of flattened or cuboidal cells with oval or rounded nucleus (Figs.1 D, E).

**Four-day embryo**

The primordium of the compound proventricular glands appeared as evaginations of the lining epithelium. Near the middle of the mesenchymal layer, the condensation of the mesenchyme was clearly observed wherein some mesenchymal cells differentiated into myoblast cells forming the prospective smooth muscle layer. Furthermore, the mesothelium became wholly flattened (Figs.1 F, G).

**Five-day embryo**

The special arrangement of the glandular epithelium became clearer and the gland primordium projected more deeply into the underlying mesenchyme. The mesenchymal cells were condensed under the epithelium of the gland rudiments and became somewhat flattened (Figs.1 H, I).

**Six-day embryo**

The proventricular glands took the simple alveolar profile consisting of a spherical tip and a stalk representing the future primary duct. The apical portion of most of the glandular epithelium became elongated, slender, and protruded into the lumen beyond the point of cell junctions, giving the epithelium a serrated appearance (Figs.1 J, K).

**Seven-day embryo**

The glands elongated without branching. The elongation of the apical portion of the glandular epithelium became evident forming profuse finger-like projections bulged into the lumen and some of them sloughed off (Figs.1 L, M).

**Eight-day embryo**

The proventricular mucosal surface showed many small folds (plicae) with intervening depressions (sulci). The proventricular glands began to be branched. Most of the glandular epithelium became simple columnar in type, whereas the pseudostratified columnar epithelium was observed in small areas. The muscular coat was differentiated into lamina muscularis mucosae and tunica muscularis (Figs.1 N, O).

**Nine-day embryo**

The proventricular glands' branching increased. Sloughing of the apical portion of the glandular epithelium became greater, and some of the epithelial cells lining the glands as well as the ducts transformed into low columnar cells (Figs.1 P, Q).

**
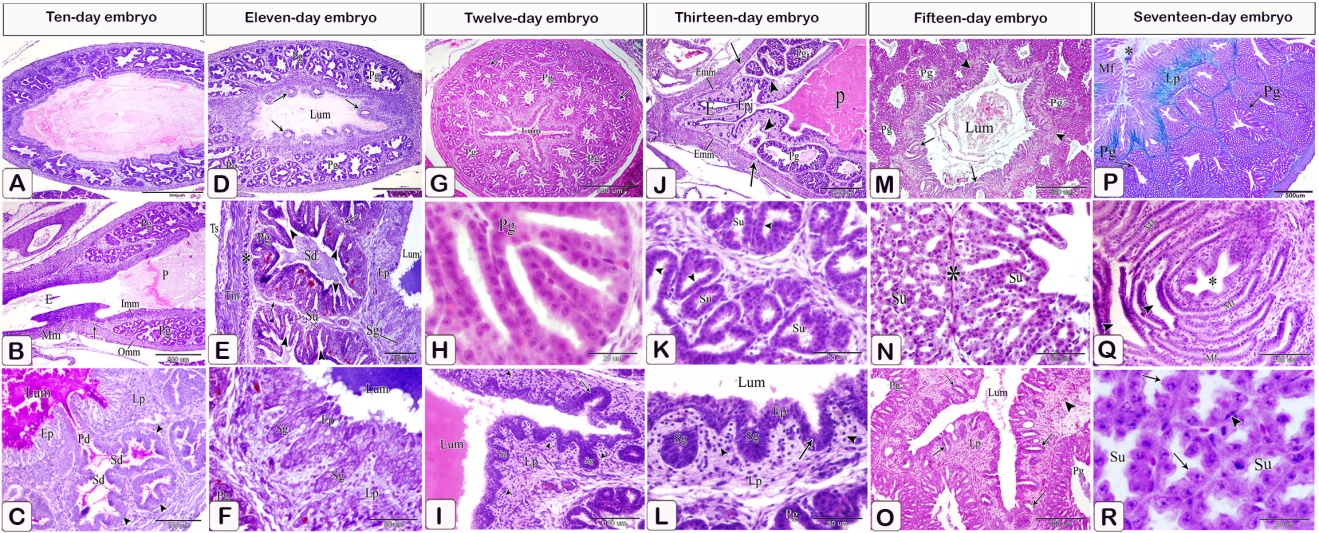
**

**Figure 2:**

**Ten-day embryo**

The primary duct of the proventricular gland is divided into two secondary ducts connecting it with the secretory units. The muscularis mucosae of the esophagus diverged at the esophago-proventricular junction into the inner and outer layers, which continued with the inner and outer layers of the muscularis mucosae of the proventriculus (Figs2. A, B, C).

**Eleven-day embryo**

The mucosal folds became more developed and forming the mucosal papillae. The proventricular glands forming the thickest portion of the proventricular wall. The primordium of the simple tubular glands could be detected as localized down-growths of the luminal epithelial cells into the lamina propria forming solid cords (Figs2. D, E, F).

**Twelve-day embryo**

The compound tubuloalveolar glands became more branched and most of their lining epithelium was of low columnar type. The connective tissue septa separating the gland lobules became thinner .The simple tubular glands increased in number and very few of them began to be canalized (Figs2. G, H, I).

**Thirteen-day embryo**

Some simple tubular glands became more canalized. The inner and outer layers of the muscularis mucosae re-joined prior to the isthmus. The mucosal folds became longer, lined with simple columnar epithelium, and had a connective tissue core of lamina propria. The lining epithelium of the compound glands' secretory units was composed of low columnar cells with few cuboidal cells interspersed (Figs2. J, K, L).

**Fifteen-day embryo**

The tip of the mucosal fold was lined with simple columnar epithelium, which transformed into simple cuboidal toward the base of the fold. In some areas, additional folds and sulci were arranged concentrically around the glandular duct opening, giving the mucosal papilla a spiral appearance (Figs.2 M).Many of the simple tubular glands showed complete canalization, lined with simple cuboidal epithelium and opened into the base of the sulci between the mucosal folds. They were located in the lamina propria, superficial to the discontinuous inner layer of the muscularis mucosae (Figs.2 O). The secretory units of the compound glands became lined solely with simple cuboidal epithelium (oxyntico-peptic cells), and the apical portion of the cells appeared regular. The lumina of the secretory units were free from cellular debris (Fig.2 N).

**Seventeen-day embryo**

At this age, the four tunics constituting the wall of the proventriculus became well established as tunica mucosa, very thin tunica submucosa, tunica muscularis, and outer most tunica serosa. The two types of proventricular glands situated within the tunica mucosa. The compound proventricular glands became much more branched, forming most of the thickness of the wall. The gland lobules were closely packed together and separated only by a very thin layer of connective tissue containing smooth muscle fibers, which extended to surround the secretory units. The light and scanning electron microscopic results revealed that more folds were added to the concentric plical structure of the mucosal papilla, giving it a definite whorled appearance (Figs.2 P,Q, R).

**Semi-thin Selection details**

They were rinsed four times for 15 minutes in a 0.1-M sodium phosphate buffer (pH 7.2), then post-fixed for two hours at 4C with 1 percent osmic acid in a 0.1-M sodium phosphate buffer. The materials were dehydrated using graded ethanol followed by propylene oxide after being rinsed three times for 20 minutes in a 0.1-M phosphate buffer (pH 7.2). In an ascending graded sequence of ethanol (50 percent (for 30 minutes), 70 percent (overnight), 90 percent (for 30 minutes), 100 percent I (for 30 minutes), and 100 percent II (for 30 minutes), samples were dehydrated (for 60 min). The samples were dehydrated. In an ascending graded sequence of ethanol (50 percent (for 30 minutes), 70 percent (overnight), 90 percent (for 30 minutes), 100 percent I (for 30 minutes), and 100 percent II (for 30 minutes), samples were dehydrated (for 60 min). The resin was used to implant the dehydrated samples Propylene oxide (Merck, Darmstadt, Germany) for 30 minutes, Epon–propylene oxide (ratio, 1:1) for 30 minutes (Epon–Araldite After that, Epon for 3 hours. Epon was made (5 mL Epon812 (Polysciences Eppelheim, Germany) + 5-ml Araldite + 12-ml dodecenylsuccinic acid hydroxide (DSAA). An incubator was used to completely combine Epon60-degree shaker The Epon mixture and an accelerator (2,4,6-Tris[dimethylaminomethyl]phenol; 1.5 percent, DMP30) were used to polymerize the samples (1.5 percent ). The blocks were incubated for three days at 60 degrees Celsius on the first day, 70 degrees Celsius on the second day, and 75 degrees Celsius on the third day.
